# Osteochondral Tissue Engineering: The Potential of Electrospinning and Additive Manufacturing

**DOI:** 10.3390/pharmaceutics13070983

**Published:** 2021-06-29

**Authors:** Andreia M. Gonçalves, Anabela Moreira, Achim Weber, Gareth R. Williams, Pedro F. Costa

**Affiliations:** 1 BIOFABICS, Rua Alfredo Allen 455, 4200-135 Porto, Portugal; andreia.goncalves@biofabics.com (A.M.G.); anabela.moreira@biofabics.com (A.M.); 2 Fraunhofer Institute for Interfacial Engineering and Biotechnology IGB, Nobelstrasse 12, 70569 Stuttgart, Germany; achim.weber@igb.fraunhofer.de; 3 UCL School of Pharmacy, University College London, 29-39 Brunswick Square, London WC1N 1AX, UK; g.williams@ucl.ac.uk

**Keywords:** osteochondral defect, electrospinning, additive manufacturing, bioreactors, induced pluripotent stem cells

## Abstract

The socioeconomic impact of osteochondral (OC) damage has been increasing steadily over time in the global population, and the promise of tissue engineering in generating biomimetic tissues replicating the physiological OC environment and architecture has been falling short of its projected potential. The most recent advances in OC tissue engineering are summarised in this work, with a focus on electrospun and 3D printed biomaterials combined with stem cells and biochemical stimuli, to identify what is causing this pitfall between the bench and the patients’ bedside. Even though significant progress has been achieved in electrospinning, 3D-(bio)printing, and induced pluripotent stem cell (iPSC) technologies, it is still challenging to artificially emulate the OC interface and achieve complete regeneration of bone and cartilage tissues. Their intricate architecture and the need for tight spatiotemporal control of cellular and biochemical cues hinder the attainment of long-term functional integration of tissue-engineered constructs. Moreover, this complexity and the high variability in experimental conditions used in different studies undermine the scalability and reproducibility of prospective regenerative medicine solutions. It is clear that further development of standardised, integrative, and economically viable methods regarding scaffold production, cell selection, and additional biochemical and biomechanical stimulation is likely to be the key to accelerate the clinical translation and fill the gap in OC treatment.

## 1. Introduction

### 1.1. Osteochondral Damage: Current Challenges

Synovial or diarthrodial joints comprise most of the body’s articulations and are characterised by wide ranges of near-frictionless motion, as well as load transferring capabilities during weight-bearing [1]. In these joints, two articulating bones glide smoothly through each other due to the presence of a composite system known as the osteochondral (OC) unit. This unit is composed of articular cartilage (AC) covering the bone surfaces, an interface layer of calcified cartilage, and the underlying subchondral bone (SB) [2,3]. Whilst AC is a highly specialised connective tissue with lubricant, wear-resistant and shock-absorbing functions that facilitates the transmission of compressive, tensile and shear loads onto the underlying bone [4], SB is responsible for distributing mechanical loads across the joint surface, thus supporting the overlying AC and ensuring stable motion [5].

In normal conditions, there is healthy homeostatic crosstalk between cartilage and bone that leads to regulated tissue remodelling and joint integrity maintenance [6]. This regulation is achieved by tightly controlled cellular, biochemical, and biomechanical processes that ensure the physiological behaviour of the human chondro-osseous junction [7]. Nevertheless, upon any damage, joint homeostasis is disturbed, and a catabolic unhealthy crosstalk is developed, leading to dysregulated bone remodelling, imbalanced cartilage regulation and progressive OC degeneration [8]. Traumatic injuries and degenerative diseases (usually associated with ageing, inflammatory disorders and underlying genetic predisposition) account for the most common causes of OC damage [9,10].

### 1.2. Aetiology and Epidemiology: Trauma vs. Degenerative Diseases

Localised OC lesions can be created acutely or developed as a result of repetitive overloading or long-term mechanical wear, with the most common OC injuries happening in the weight-bearing joints of the lower extremity (hips, knees and ankles) [9,11,12]. Such injuries are typically associated with sports activity, traumatic accidents, osteonecrosis, or osteochondritis dissecans, and therefore affect greatly the active population, including high-level athletes [13,14]. In fact, chondral and OC defects are observed in 20 to 60% of all anterior cruciate ligament ruptures—a highly common injury among athletes [15]—and the prevalence of full-thickness chondral defects of the knee in athletes was found to be 36% in a cohort study involving 931 individuals, with a mean age of 33 years old [16,17]. Usually, during acute trauma, compressive or shear forces lead to the separation of the radial and calcified layers of cartilage, forming well-delineated focal defects that range from a simple contusion of the AC and SB to a fracture involving AC, vascularised SB and subchondral marrow [18,19]. Depending on the severity of the lesion, these might lead to progressive tissue degeneration and loss of joint function, as well as to the development of posttraumatic arthritis, which is mainly related to cartilage’s avascular nature and limited ability for spontaneous regeneration and self-repair [20].

Degenerative disorders, such as osteoarthritis (OA), osteoporosis, rheumatic arthritis, and other musculoskeletal disorders, also play an important role in the development of OC lesions and subsequent joint failure. OA, the most common form of arthritis, is a chronic disease of the articular joint characterised by the degeneration of AC and malfunction of the affected joint [21,22]. Approximately 250 million people are suffering from OA worldwide [23], of whom 30% are over 60 years of age and present substantial mobility limitations [24]. These numbers reflect the high socioeconomic burden of OC defects, which can cost between 1–2.5% of a country’s gross domestic product (GDP) [25].

### 1.3. Current Therapies: The Pros and Cons

Non-operative treatment of OC lesions is usually the primary course of action for minor OC defects and includes the use of chondroprotective pharmacotherapy (e.g., intra-articular hyaluronic acid, steroid or platelet-rich plasma (PRP) injections), the administration of non-steroidal anti-inflammatory drugs (NSAIDs) to fight pain and inflammation, or physiotherapy [26,27,28]. In contrast, when there is greater damage to the joint, including detached cartilage and/or bone fragments, surgical procedures are usually applied to restore OC structure and function. These can be divided into palliative, reparative, and restorative treatments [29]. Palliative methods (such as arthroscopic lavage and debridement, abrasion arthroplasty and chondroplasty) do not intend to replace defective OC tissue, but instead, provide relief of symptoms. Reparative and restorative treatments aim to reconstruct the defective area and to repair or regenerate damaged OC tissue, respectively and improve joint functionality [26,30].

Reparative methods include microfracture and drilling, as well as autologous or allogeneic OC transplantation (mosaicplasty). Microfracture and drilling are bone marrow stimulating techniques used to initiate OC regeneration [31]. These techniques seek spontaneous and natural healing of the AC by perforating through the SB to promote bleeding into the lesion site and subsequent recruitment of marrow-derived mesenchymal stem cells (MSCs) and bioactive molecules, which might increase cartilage repair capacity [32]. Nonetheless, this procedure often culminates in the formation of fibrocartilage that exhibits reduced resilience and stiffness, higher permeability and poorer load-bearing capacity when compared to natural hyaline cartilage, making it unable to withstand physiological loading. Hence, despite being relatively minimally invasive, presenting a short surgery and recovery time, and showing positive short-term clinical outcomes, microfracture has high inter-patient variability and is not very effective in promoting OC damage restoration in the long run [33,34,35].

OC graft transplantation is a more invasive reparative method that is based on the application of OC grafts of autologous (originated from the patient) or allogeneic (derived from a matching donor) origin [36]. Despite the promising use of both OC graft transplantation techniques to replace defective OC tissue, each has inherent advantages and disadvantages. Compared to autografts, OC allografts are not associated with donor-site morbidity or the need to undergo multiple surgical procedures and can be applied to reconstruct extensive lesions (>10 cm^2^) [37,38]. However, they are also linked to possible disease transmission, immunogenicity, slower tissue remodelling, poor integration into the lesion site and difficulty in preservation methods (including maintaining chondrocyte viability and tissue biochemical, biomechanical and functional properties for prolonged periods), which is avoided when using autografts [39,40,41]. Despite their limitations, there are some successful examples of allogeneic OC grafts currently being applied in the clinic [42,43]. Chondrofix^®^ allograft (Zimmer Biomet, Warsaw, IN, USA) is the first off-the-shelf OC allograft available since 2012. It combines donated human decellularised hyaline cartilage and cancellous bone and is indicated for the treatment of severe OC lesions in a single, low-invasive, procedure [43,44]. Furthermore, DeNovo^®^ NT (Zimmer Biomet, USA) is a Food and Drug Administration (FDA)-listed tissue product used for joint cartilage repair [45]. It uses allogeneic AC harvested from juvenile donors and divided into chondral fragments that are secured into focal cartilage defects in a single-stage procedure using fibrin adhesive [43,46].

Lastly, biological replacement techniques using cultured autologous chondrocytes have also been used as chondral and OC restorative treatment alternatives [47]. Autologous chondrocyte implantation (ACI) was first reported in 1994 by Peterson and Brittberg as a pioneering treatment for chondral knee lesions [48]. This technique involves cartilage harvesting, followed by the implantation of cultured autologous chondrocytes under an autologous periosteal flap. Different generations of ACI protocols have arisen since its first development to overcome one of its major limitations: cell retention [49,50,51]. In one of these advanced ACI methods, autologously isolated and enriched chondrocytes are combined with a synthetic type-I/III collagen matrix during the culturing process—this is termed matrix-induced autologous chondrocyte implantation (MACI) [52,53]. The use of a three-dimensional (3D) supporting matrix allows optimisation from both the biological and surgical point of view, since it helps even the distribution of chondrocytes in the transplanted site and avoids the need for highly invasive procedures. Both biological strategies are currently being used as a treatment for symptomatic chondral and OC defects (especially in damaged knee joints of young patients), yet their application is still controversial compared to previously mentioned therapies such as microfracture or mosaicplasty [54,55]. Moreover, ACI still exhibits critical disadvantages that have hampered their wide use in the orthopaedic field. Besides intrinsic limitations including the use of insufficient cell numbers, uncontrolled cell differentiation, immunogenicity, unsatisfactory integration of the de novo tissue into the host OC unit, and failure to prevent fibrocartilaginous healing (native healthy AC is still unmatched by any available product), these methods require two surgical procedures, have relatively long recovery times, and are typically rather invasive [56]. It is also important to stress that these therapies are not curative, which means they can only delay the progress of OC tissue degeneration and will eventually culminate in the need for a total joint replacement surgery as an end-stage intervention. Alas, this medical procedure is itself associated with several disadvantages, including the need for extremely invasive surgery, infection, abnormal wear, implant irritation and pain, and limited lifespan (~10–20 years), after which a revision surgery might be required [57].

### 1.4. The Need for Improved Osteochondral Regenerative Solutions

Despite the promising results of current therapies, most focus on conservative treatments and no definitive and consensual solution has been proven to ensure complete and long-lasting functional repair and regeneration of bone and cartilage tissues [3,35,58]. The problems encountered are mainly related to the biological, biochemical and biomechanical properties of the whole OC unit, which is typically exposed to high pressure and motion and presents limited healing potential due to the poor regenerative capacity of AC and its complex interaction with the underlying SB [19,38,59]. Additionally, the decrease in the number and proliferative capacity of endogenous stem cells and tissue regenerative capacity with ageing hinders the search for therapeutic solutions. Therefore, due to the enormous socioeconomic burden of OC-related problems and the lack of suitable long-term therapeutic solutions [19], the development of improved and innovative treatments capable of promoting OC tissue regeneration is imperative.

Given the promising role of tissue engineering (TE) in the development of biomimetic OC tissue constructs (that is, those capable of replicating the native conditions of bone and cartilage environments), the present study reviews the osteochondral tissue engineering (OCTE) state-of-the-art, with emphasis on (1) cutting-edge preparation methodologies, namely electrospinning and 3D printing; and (2) the most promising cell sources and biochemical stimuli used to regenerate OC tissue. A critical perspective on the different elements of the tissue-engineered OC constructs is applied to understand what is preventing their translation to the clinical setting, and the most pressing requirements needed to bring OCTE back on track and closer to the patients are identified. Furthermore, relevant strategies to standardize ongoing research and streamline the choice in terms of scaffold material, biofabrication methods, cell type and biochemical stimuli are suggested.

## 2. Osteochondral Unit: Composition, Structure, and Function

The development of innovative therapeutic solutions capable of promoting the functional repair and regeneration of damaged OC tissue requires an understanding of the specific hierarchical structure and biological properties of cartilage and bone, as well as the crosstalk established within the OC unit under physiologic and pathologic conditions. Additionally, the identification of the key requirements to replicate these mechanically, physiologically and biochemically interdependent tissues in vitro is of critical importance to achieving candidates able to improve the current clinical scenario.

### 2.1. Articular Cartilage

AC is a type of hyaline cartilage that covers the bones’ articular surface, forming a thin layer of highly specialised connective tissue [4,60]. AC is avascular, aneural and alymphatic in nature, and it is composed of a dense ECM with a sparse distribution of a single type of specialised cells called chondrocytes [4,60,61]. Chondrocytes account for 1–5% of the total cartilage volume and are responsible for the synthesis, organisation and maintenance of cartilaginous ECM [61,62]. Mature chondrocytes display no detectable mitotic activity and are known to maintain a balanced metabolism that creates an equilibrium between anabolic and catabolic processes [2,63]. The direction of this balance is regulated, among other factors, by the mechanical loading of cartilage through mechano-transduction pathways [64]. Hence, in response to damage, local chondrocytes can detect the changes occurring within the matrix and react accordingly [62,65].

Regarding ECM composition, two phases can be distinguished: a fluid phase, composed mostly of water (65–85% of the total wet weight) and a solid organic matrix, which is mainly composed of collagens (60–70% of the total dry weight) and proteoglycans (around 30% of the total dry weight), known to provide tensile strength and compressive resilience, respectively [4,5].Collagen type II, the major organic component of AC, represents 90–95% of the collagen content and forms highly organised networks of crosslinked fibrils that constrain proteoglycan aggregates and interact with other collagens (including types VI, IX, X and XI), small proteoglycans, and other matrix proteins [2,4]. In hyaline cartilage, multiple aggrecan molecules are non-covalently bound to a long hyaluronic acid chain, forming large proteoglycan aggregates [5,66]. Given their high negative charge, these structures are responsible for water uptake, essential for ensuring the stability of the tissue during stress compression and release, as well as for osmolarity maintenance [66].

**Figure 1:**
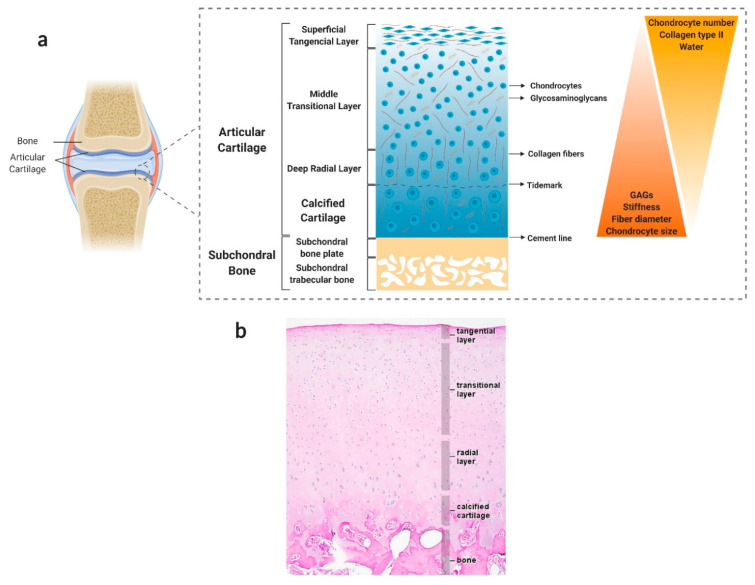
Hierarchical structure, composition, and properties of the native osteochondral (OC) unit. (**a**) Schematic illustration of the OC unit. This multi-tissue region can be divided into three major layers: articular cartilage (AC), which consists of hyaline cartilage tissue; calcified cartilage, which marks the transition from soft cartilage to stiff subchondral bone (SB); and SB, which can be divided into two anatomically distinct layers, the SB plate (cortical-like bone) and the subarticular spongiosa (trabecular-like bone). AC can itself be split into four sublayers based on collagen fibre alignment, proteoglycan composition and chondrocyte number and morphology. From top to bottom, these are the superficial zone, the middle zone, the deep zone, and the calcified zone. Created with BioRender.com (accessed on 30 March 2021). (**b**) Representative histological image of the OC unit. Adapted from [84] with permission from Elsevier. Copyright © 2009, Elsevier Ltd.

As illustrated in Figure 1, AC can be divided into four structurally different layers: the superficial tangential layer (10–20% of AC thickness), the middle transitional layer (40–60% of AC thickness), the deep radial layer (30–40% of AC thickness), and the calcified layer [4,67,68]. These layers differ in cellularity, cell morphology, matrix composition (e.g., collagen fibril content and orientation, the concentration of proteoglycans and water content), thickness and mechanical properties. It is this unique structure and composition of the ECM together with precise chondrocyte-matrix interactions that determine the biomechanical properties of AC under both physiologic and pathological conditions [61,62].

### 2.2. Cartilage–Bone Interface: Calcified Cartilage

The calcified cartilage is a narrow tissue layer that marks the transition from soft cartilage to stiff SB and helps convert shear stresses into compressive and tensile stresses during joint loading and kinematics [2,69,70]. Calcified cartilage is separated from the AC by a histologically defined tidemark, a thin layer metabolically active for calcification that establishes the interface between soft and calcified cartilage, therefore being considered the mineralisation/calcification front [2,71]. In homeostatic conditions, the tidemark functions as a physical barrier and allows the AC and the SB to maintain distinct physiological environments [72].

### 2.3. Subchondral Bone

Located underneath the calcified cartilage layer and separated from this by the cement line (a less pronounced border compared to the tidemark), SB is organised into two anatomically distinct structures with unique architectural, biological and mechanical properties: the SB plate and the subarticular spongiosa, which represent cortical and trabecular/cancellous bone, respectively [5,73]. SB is a highly vascularised and innervated tissue that, together with the synovial fluid, contributes as a nutritive source for AC. Indeed, microvessels from the subchondral region are thought to extend to the deepest layers of AC (radial and calcified layers), potentiating the diffusion of nutrients and small molecules [74,75].

Bone is a heterogeneous composite material made of both organic and inorganic components. Approximately 60–70% of bone net weight is mineral material, 25–30% is an organic material and 5–10% is water [76]. The mineral bone matrix is mainly composed of hydroxyapatite crystals (Ca_10_(PO_4_)_6_(OH)_2_), formed by the precipitation of calcium phosphate minerals. The remaining organic phase consists mostly of collagen type I fibrils (90–95% of the organic matrix) which comprise the basic building block of the bone matrix network, non-collagenous proteins, proteoglycans and lipids [74,77]. While the nanoscale hydroxyapatite crystals contribute to the rigidity and load-bearing strength of bone, collagen provides flexibility and elasticity to the tissue [76]. Although bone cells make up less than 2% of the bone mass, they are vital for maintaining osseous function. Four types of cells are found within bone tissue: osteoblasts (bone-forming cells), osteocytes (terminally differentiated and trapped osteoblasts), bone lining cells and osteoclasts (phagocytic cells responsible for bone resorption) [78,79].

The SB plays a crucial role in maintaining the cartilaginous environment and function by mechanically and metabolically supporting the AC, preserving the joint structure, and absorbing most of the inflicted shock [5,80]. This can be related to the inherent Young’s modulus of the tissues, since the modulus of hyaline cartilage is 0.5–2 MPa [81] and SB’s modulus is in the range of 16–23 GPa for cortical bone and approximately 1–2 GPa for trabecular bone [82,83].

As previously outlined, AC and SB exhibit considerable distinct structural, mechanical, physicochemical, and biological features spanning from the nanoscale to the macroscale [85]. These differences translate into distinct intrinsic repair and regenerative capacities: while bone has inherent regeneration potential as part of the repair process in response to injury, as well as during continuous remodelling throughout adult life, cartilage exhibits a very low endogenous healing capacity [86]. Despite this fact, the tissues in the OC unit coexist as a single functional unit, during both physiological and pathological conditions [2]. There is a close interaction between cartilage and bone that, although not completely understood, must be considered when replicating the OC junction in vitro and in vivo. Moreover, the use of interfacial tissue with a gradual variation of bone and cartilage features is an essential step when trying to obtain such tightly interconnected structures [38,85,87].

## 3. Osteochondral Tissue Engineering

Given the cellular, biochemical, structural, and mechanical requirements found for OC tissue, it has become clear that highly specialised methodologies are needed to replicate such an environment in vitro. In this regard, TE is one of the most promising strategies, since it aims to promote the formation of new functional tissues similar to their native counterparts by using 3D biomaterial scaffolds, cells, and signalling molecules (e.g., growth factors), either combined or alone [88]. Indeed, distinct tissue-engineered matrices have been shown to provide the conducive conditions necessary for cell communication with the surrounding environment, as naturally promoted in vivo by cell-cell and cell-ECM interactions, therefore showing their great potential for damage repair and tissue regeneration [89].

The successful design of cell-instructive 3D microenvironments requires that both general biological and physical criteria, as well as specific tissue features, are taken into account. Besides being biocompatible and biodegradable, the designed constructs should be able to mimic the native mechanical properties (e.g., stiffness and viscoelasticity), permeability, porosity, and overall architecture of the OC tissue. In particular, when developing tissue-engineered constructs, a compromise between the materials’ cell supportive nature and the rate of degradation must be found to ensure that cells have time to synthesize their own ECM and produce functional neo-OC tissue [86,90]. However, other critical aspects specific to bone and cartilage tissues must also be considered when replicating the OC unit in vitro.

**Figure 2:**
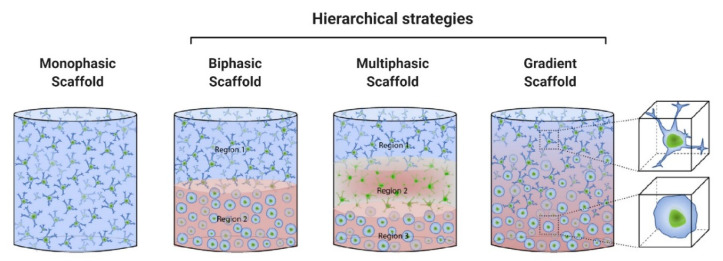
Schematic diagram of osteochondral tissue-engineered (OCTE) strategies used for the biofabrication of OC tissue replicates in vitro. Different approaches can be classified according to the number of layers and gradient properties of the designs: monophasic scaffolds are formed by a single homogeneous layer, while discrete gradient constructs can be bi- (two layers) or multiphasic (three or more layers). In the latter, each layer represents a specific region of the OC unit. Because multiphasic, discrete, scaffolds are associated with abrupt transitions between different phases, continuous gradient scaffolds have also been developed, in which a gradual transition between separate regions better emulates the native features of the joint. Adapted from [81] with permission from Elsevier. Copyright © 2016, Acta Materialia Inc. Published by Elsevier Ltd.

As previously discussed, AC and SB have a distinctive hierarchical structure and biological properties which translate into unique biomechanical abilities. Hence, single homogeneous scaffolds have difficulties meeting the full complexity of the chondro-osseous junction tissue, and so hierarchical scaffolds with gradient physical and chemical properties are essential to produce smooth transitions between skeletal tissues with significant differences. The most successful OC tissue-engineered designs are based on bi-/multi-layered matrices that exhibit a discrete gradient of biochemical, structural and mechanical features, or matrices with a continuous gradient of properties, where there is no distinct interface between each layer [86,91]. This is schematically illustrated in Figure 2. While discrete gradient scaffolds are fabricated by integrating individual phases into a single construct by suturing, glueing, or press-fitting, continuous gradient scaffolds do not exhibit individual layers and are instead fabricated as a single matrix with gradient properties [91,92,93]. This is especially relevant since discrete gradient scaffolds are known to show abrupt and substantial changes in terms of the structural and mechanical properties of the different phases, which is often associated with layer delamination and tissue separation upon loading [81,91].

Regardless of the chosen design strategy, effective OC constructs need to have chondral- and osteo-mimicking regions, with properties similar to those of cartilage and bone tissue, respectively. While the chondral section should resemble the native AC with its dense ECM of collagen type II fibres and hyaluronic acid molecules organised along the different AC sublayers, the osseous section should replicate the complex micro/nano anatomical bone structure including the nanoscale hydroxyapatite composites deposited along the collagen type I bundles. Such organisation should provide the precise matrix porosity, permeability, and stiffness needed for neocartilage and neobone tissue growth, whilst ensuring functional host integration in vivo. Bearing this in mind, scaffolding materials such as porous structures, fibrous networks and hydrogels that can accurately model the complexity of the OC unit are important prospective candidates for OC tissue repair and regeneration [90].

### 3.1. The Building Blocks of an Osteochondral Tissue-Engineered Construct

#### 3.1.1. Biomaterials

The extensive research in the field of OC regeneration has led to the identification of a plethora of biomaterials, with a wide range of chemical compositions and mechanical properties, which have provided promising templates for successful OCTE strategies. These matrices may consist of naturally or synthetically produced materials and encompass both organic and inorganic components, therefore effectively mimicking the native structure of the OC unit. However, such an ample spectrum of possibilities requires a careful evaluation of the characteristics of each available material, in order to identify whether its properties meet the abovementioned requirements of an OC tissue-engineered scaffold.

Materials derived from natural resources previously used in OC constructs include alginate, chitosan, gelatine, silk fibroin, and native bone or cartilage constituents like collagen and GAGs [94]. Chitosan and alginate have raised particular interest in this context, owing to their polysaccharide chemical nature emulating the GAGs present in cartilaginous ECM, including hyaluronic acid and chondroitin sulphate [94,95]. Gelatine is another attractive naturally occurring polymer since it derives from collagen and has a denatured structure that decreases the possibility for immunogenicity in allogeneic or xenogeneic transplantation [94]. In turn, silk fibroin is an amphiphilic, biocompatible polymer that presents adjustable degradation rates and suitable mechanical properties for use in OC constructs [96]. Most commonly, several of these biomaterials are integrated into a single OCTE strategy, so as to combine their physicochemical properties and attain a more robust construct capable of mimicking both AC and SB layers. Blends of chitosan-alginate [95,97,98], chitosan-silk fibroin [99,100], and alginate-hyaluronan [101,102,103], for example, have been used for the fabrication of OC scaffolds. In several reports, these organic polymers were further combined with ceramics like β-tricalcium phosphate (β-TCP) [97], polyphosphate [98], hydroxyapatite [99,100,102,103,104] or bioglass [105], thereby resembling the inorganic phase present in SB.

Decellularised extracellular matrices (dECMs) have been gaining traction over the last few years in TE, being derived from natural tissues or organs which have been depleted of cellular components yet maintain the structural architecture and ECM constituents of the native tissue [106]. As pointed out by Taylor et al. [107], decellularised matrices display the properties of an ideal scaffold: exclusive tissue-specific architecture, vascular networks, and an intricate composition and structural organisation that is difficult to achieve with artificial manufacturing. dECMs efficiently support cell adhesion and proliferation, since they are endowed with topographical and biochemical cues that stimulate cellular responses [108]. As such, many studies have already been performed for the application of decellularised matrices for bone and cartilage TE (comprehensive reviews can be found in [108,109]). Importantly, in vivo implantation of dECM-based scaffolds in surgically induced rabbit OC defects led to good graft integration, production of hyaline cartilage ECM constituents like GAGs and type II collagen, and overall accelerated defect filling and healing [110,111].

However, a frequent problem associated with the use of whole cartilage dECM is the elevated density of the matrix that hinders the migration of endogenous or exogenously seeded cells to the interior of the material, hampering tissue remodelling and graft integration [112,113]. In addition, the decellularization process often affects the biochemical and biomechanical properties of the matrix, therefore compromising its functionality. In fact, an observation that is quite recurrent throughout the literature is that conventional decellularization protocols preserve the collagen ECM structure, but markedly affect the GAG content [112,113,114,115,116]. This may contribute to a less dense matrix and favour cell migration towards the interior of the scaffold, but it often results in poorer biomechanical and load-bearing properties. Other disadvantages associated with the use of dECMs in TE are applicable to natural biomaterials in general: the weak mechanical attributes of naturally occurring materials and their associated batch-to-batch variability are further discouraging factors [88]. Besides, serious complications may arise from scaffold immunogenicity and potential disease transmission as often happens when allografts or xenografts are introduced as treatment solutions.

As a promising alternative, synthetic polymer production has created an ever-growing library of new biocompatible compounds with highly tuneable physicochemical properties, enabling the development of a large number of tissue-engineered constructs with varying hydrophilicity, porosity, mechanical strength, and degradation rates. Indeed, the FDA has already approved a range of synthetic polymers for medical applications. In the context of OCTE, several of these synthetic polymers have been used for the fabrication of bone and cartilage scaffolds, either individually or in composite structures. Poly(lactic-*co*-glycolic acid) (PLGA) is one of the most frequently used synthetic polymers in TE, due to its highly tuneable degradation rates and excellent biocompatibility [117]. PLGA porous scaffolds have demonstrated the ability to support chondrocyte survival, proliferation, and ECM deposition [118]. Poly(ε-caprolactone) (PCL) is another polyester that has been widely utilised for bone and cartilage regeneration, owing to its favourable physicochemical and mechanical properties, but its intrinsic hydrophobicity makes it unsuitable for the promotion of cell adhesion and proliferation [119]. Combination of PCL scaffolds with other materials, such as a PLGA-poly(ethylene glycol) (PEG)-PLGA copolymer hydrogel [120], graphene [121,122], or hydroxyapatite [123], may boost the performance of the PCL backbone and result in enhanced cell adhesion, proliferation, and ECM deposition.

Besides polyesters, hydrogels composed of poly(ethylene glycol) (PEG) have been proved capable of promoting chondrocyte growth and cartilage ECM production [124,125]. It is worth noting that PEG is not only biocompatible and bioinert but also highly hydrophilic, forming constructs with water content close to that of soft tissues [126]. A few reports have also proposed the use of polydimethylsiloxane (PDMS) for the fabrication of OC scaffolds, in which both human MSCs [127] and adipose-derived stem cells (ASCs) [128] could be cultured and the expression of osteogenic markers (alkaline phosphatase (ALP), calcium deposition) was promoted, especially when this material was coated with reduced graphene oxide [128]. Similarly, polyacrylate-based materials support chondrocyte cell attachment and production of GAGs and collagen, and their subcutaneous implantation in mice demonstrated good integration with the native tissue and no signs of inflammatory response [129]. In addition, a recent study has shown that the mechanical properties of AC, namely its swelling behaviour, can be mimicked by composite gels of poly(acrylic acid) microparticles embedded within poly(vinyl alcohol) (PVA) matrices [130]. As a final example, polyurethane (PU)-based scaffolds have demonstrated satisfactory mechanical properties and the ability to sustain chondrocyte and MSC proliferation and ECM deposition [131], even though in vivo studies in an equine OC defect model showed very limited efficacy of a PU elastomer in promoting hyaline cartilage formation and tissue regeneration [132].

Synthetically produced polymers have the advantage of enabling precise control over their structure, mechanical properties, and chemical compositions, which is associated with higher reproducibility and decreased variation between batches [133]. In spite of this, synthetic biomaterials have a very important shortcoming: their artificial and bioinert nature hinders cell recognition and attachment, due to the absence of natural ECM components that can guide these processes and enhance neotissue formation and organisation. This drawback has led to a growing number of studies focusing on the development of composites or blends containing both synthetic and naturally occurring elements so that the reproducibility and mechanical properties of artificial materials can be combined with cell-instructive ECM constituents [134,135,136,137].

In summary, and taking the information presented above into account, a few essential factors need to be kept in mind when selecting biomaterials for OCTE. While it is important to include natural bone and cartilage ECM components, in order to elicit cell recognition, attachment, and cell-mediated tissue remodelling, all-natural constructs tend to be associated with limited reproducibility, insufficient mechanical properties, and potential immunogenicity. Synthetic polymers are a valuable solution for these issues; alas, they lack cell-guiding properties. Accordingly, hybrid strategies have emerged as encouraging alternatives for OC treatment, where the benefits and the caveats of naturally and artificially produced materials conveniently complement each other. Importantly, the success of an OC construct rests not only on its biochemical composition and biomechanical properties, but also on its topography and architecture, as this structural organisation will potentially dictate cell adhesion, proliferation, migration, and differentiation. Thus, an ideal scaffold for the treatment of OC lesions will be composed of cell-instructive cues embedded within a specialised biomimetic architecture in which osteogenesis and chondrogenesis can take place, to ensure tissue remodelling, defect regeneration, and effective functional recovery.

#### 3.1.2. Incorporation of Biochemical Stimuli

Even though the biomaterials composing an OC scaffold are the very foundations of the construct, they often need complementary elements capable of improving tissue response, integration, and repair. Most frequently, these elements consist of biochemical factors, such as chemotactic and growth factors or small molecule-based drugs (Table 1) that can trigger the homing and appropriate response of endogenous cells after transplantation, or cellular components.

##### Growth Factor Delivery

Growth factor delivery is one of the most widely used techniques to improve an OC scaffold’s bioactivity, based on the idea of recapitulating biological signalling cascades that participate in cell recruitment and homing, proliferation, and differentiation, thus directing the physiological response to tissue repair [138]. Alas, direct administration (e.g., via intra-articular injection) of growth factors is markedly ineffective, since these protein molecules have high clearance rates and, therefore, very short half-lives [139]. Consequently, growth factors must be administered frequently and in high doses to achieve a therapeutic effect, causing unwanted and dangerous side effects, including cancer [140], and decreasing patient compliance and treatment efficacy [138]. Efficient delivery of protein therapeutics is remarkably challenging, which has motivated the development of delivery systems capable of maintaining protein bioactivity and providing temporal and spatial control of protein release.

In the context of OCTE, much effort has been employed to endow engineered scaffolds with sustained protein release properties. Growth factors from the transforming growth factor (TGF)-β superfamily, including TGF-β1 [141,142], TGF-β3 [143,144], and several bone morphogenetic proteins (BMP-2 [145,146], BMP-4 [147], BMP-7 [148]), as well as insulin-like growth factor (IGF)-1 [149], basic fibroblast growth factor (bFGF or FGF2) [150], and chemokines like stromal cell-derived growth factor 1α (SDF-1α) [151], have been incorporated into OC constructs, due to their well-known physiological role in bone and cartilage tissue repair [138,152].

It should be kept in mind that the healing process in biological systems is extraordinarily complex, relying on the combined action of numerous signalling molecules that orchestrate cell migration, differentiation, and the overall homeostatic recovery. Accordingly, recent studies have also been adopting combined therapy strategies, in which multiple growth factors are delivered from a single tissue-engineered scaffold [153,154]. Such combinatory approaches may be of particular interest for OCTE, enabling the simultaneous delivery of factors involved in both SB and AC repair [155,156,157], even though obtaining positive results with dual growth factor delivery may not be straightforward [158,159]. An issue behind these combined therapy approaches is the difficulty in controlling growth factor release, so as to provide therapeutic dosages in an appropriate time frame for the promotion of cell homing, differentiation, and SB/AC tissue remodelling. In fact, growth factor doses vary over an incredibly wide range among different reports, and this lack of standardisation and consensus is problematic in the context of study reproducibility and the consistency of the obtained results [139]. Another problem lies in the fact that distinct growth factors play specific roles at different stages of OC lesion repair, therefore requiring differential release profiles with tight temporal and spatial control. Thus, further optimisation is required to achieve tuneable, but reproducible, growth factor delivery systems, capable of triggering both cartilage- and bone-reparative mechanisms towards a balanced and robust OC regenerative process.

It may also be beneficial to deliver a cocktail of active components in a single intervention, rather than any individual growth factors. Exosomes, for instance, are extracellular vesicles secreted by multiple cell types that carry a variety of biomolecules, including nucleic acids, proteins, and lipids, and guide biological processes by acting as mediators of intercellular communication. The roles of exosomes in the pathogenesis of OA, as well as their therapeutic potential, have been recently reviewed elsewhere [160]. Another promising alternative is autologous PRP, which consists of an enriched medium containing a myriad of growth factors, including TGF-β, bFGF, IGF-1, epidermal growth factor (EGF), vascular endothelial growth factor (VEGF) and platelet-derived growth factor (PDGF), together with chemokines, immune mediators, and adhesion proteins [161,162]. Abundant preclinical evidence has demonstrated that treatment of AC lesions with PRP may result in improved chondrocyte matrix production and minimised catabolism, enhanced stem cell homing, higher differentiation of stem cells into chondrogenic phenotypes, and decreased inflammation [163]. More importantly, such beneficial effects have also been confirmed by numerous clinical studies [164,165]. Additionally, the combination of PRP treatment with OC scaffolds has already been explored in vitro [166] and in vivo [167,168]. Unfortunately, these studies are also affected by a severe lack of uniformity and standardisation, both in the composition of the PRP used (which is, naturally, associated with high interindividual variability) and in the preparation of this platelet-enriched cocktail [169]. This not only impairs the attainment of reproducible results but also makes comparisons between distinct studies very difficult, explaining why the effects of PRP in OC therapy remain a controversial topic.

##### Gene Therapy

The difficulty in delivering protein-based agents in a controlled manner while also preserving their structural integrity and functional activity has led to the development of gene therapy strategies, due to the enhanced stability of DNA when compared to proteins. Importantly, gene delivery may also allow for a more effective site-specific and prolonged action [170]. The considerations necessary for efficient and successful gene therapy in the context of OCTE are outside the scope of this review but are discussed in detail elsewhere [170,171]. Although in a great number of studies gene therapy is performed by directly administering the genes of interest at the lesion site, scaffold-based gene delivery may prove advantageous [171]: not only does it provide a more tuneable release with temporal control, but it also allows for spatial distribution of osteogenic and chondrogenic genes that help achieve zonal differentiation of progenitor cells and well-defined osseous and cartilaginous layers. The most common genes used for OC gene therapy include those coding for growth factors, such as BMP-2 and TGF-β3 [172,173,174], transcription factors like Sox9 [172,175], and anti-inflammatory molecules, such as the interleukin-1 receptor antagonist (IL-1Ra) [174].

However, gene therapy is associated with serious concerns that have to date precluded its clinical use for OCTE. Numerous gene delivery approaches rely on the utilisation of viral vectors, which, despite their increased transfection efficiency and consequently high gene expression levels, are associated with the potential for immune recognition, response, and neutralisation. Non-viral vector alternatives have been developed and are currently available, but their transfection efficiencies are much lower than those of viral vectors [170]. Moreover, the difficulties in achieving permanent transgene expression and production of relevant concentrations of the targeted proteins have also limited the effectiveness of gene therapy in OC disease management [176]. Hence, particularly extensive safety and efficacy studies need to be carried in pre-clinical and early-stage clinical trials before gene therapy can make progress as a valid therapeutic option.

##### Small Molecule Delivery

**Figure 3:**
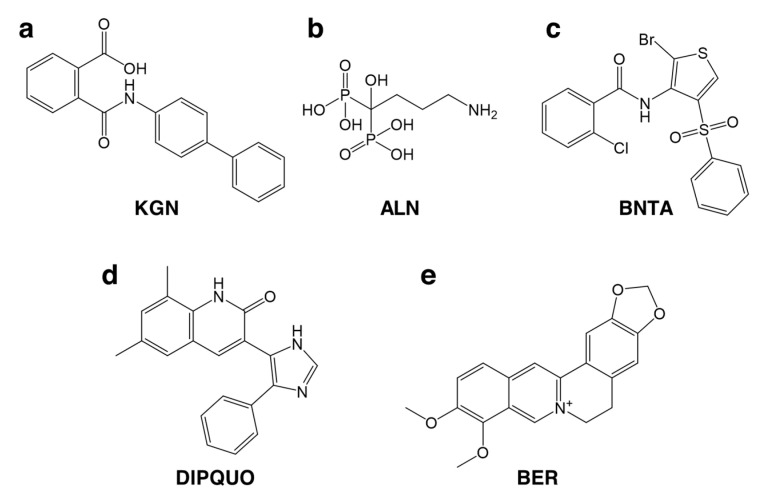
Small molecule drugs used for OC therapy. (**a**) Kartogenin (KGN). (**b**) Alendronate (ALN). (**c**) *N*-[2-bromo-4-(phenylsulfonyl)-3-thienyl]-2-chlorobenzamide (BNTA). (**d**) 6,8-dimethyl-3-(4-phenyl-1*H*-imidazol-5-yl)quinolin-2(1*H*)-one (DIPQUO). (**e**) Berberine (BER).

Small molecule drugs can also be valuable cell-instructive factors in TE. Using high-throughput screening techniques and synthetic chemistry, a never-ending variety of small molecules can be produced with high yields and relatively low cost and subsequently evaluated for safety and efficacy profiles. In addition, their effect is normally dose-dependent, allowing for a fine-tuning of their biological action, and their administration is often simpler and less challenging than that of biologics [177,178]. Therefore, a lot of research has been directed at identifying and synthesising small molecule drugs capable of inducing osteogenesis and chondrogenesis for potential OC application. A relatively recent discovery has been that of kartogenin (KGN, Figure 3a), a small molecule proven to induce chondrogenic differentiation from human bone marrow-derived mesenchymal stem cells (BMSCs) and to have chondroprotective and regenerative action in vitro and in vivo under pathological conditions associated with OA [179]. KGN can also inhibit catabolic reactions, through the upregulation of the expression of tissue inhibitors of metalloproteinases (TIMPs) and reduced expression of matrix metalloproteinases (MMPs) [180,181]. Likewise, KGN has an immunomodulatory behaviourthat has resulted in the upregulation of anti-inflammatory markers (IL-10) and higher T-cell differentiation into regulatory phenotypes (T_reg_) in mouse and rat models of OA [181]. Interestingly, exosomes derived from BMSCs pre-conditioned with KGN have enhanced chondrocyte proliferation and migration in vitro and accelerated cartilage repair after hydrogel encapsulation and implantation into rat chondral defects [182].

Liu and colleagues [183] have taken advantage of this potential of KGN in OC regeneration and combined it, in a biphasic scaffold, with alendronate (Figure 3b), a bisphosphonate shown to inhibit bone resorption by osteoclasts and potentially assist AC healing by accelerating SB regeneration [184]. Bisphosphonate therapy in OA patients has also resulted in pain alleviation, reduced stiffness, and improved functional recovery [185]. Importantly, KGN- and alendronate-loaded biphasic scaffolds were able to promote the differentiation of embedded BMSCs into both chondrogenic and osteogenic phenotypes in vitro and after subcutaneous implantation in a rat model [183].

Recently, *N*-[2-bromo-4-(phenylsulfonyl)-3-thienyl]-2-chlorobenzamide (BNTA) was identified as a potential disease-modifying OA drug [186]. BNTA (Figure 3c) was shown to stimulate cartilage ECM production and to exert a protective and regenerative effect in a rat model of trauma-induced OA. The authors demonstrated that BNTA carried such protective effects by upregulating gene and protein expression of superoxide dismutase 3 (SOD3), an antioxidant extracellular enzyme responsible for the scavenging of superoxide anions. DIPQUO (6,8-dimethyl-3-(4-phenyl-1*H*-imidazol-5-yl)quinolin-2(1*H*)-one, Figure 3d) is another novel small molecule proven to induce osteogenic differentiation of hMSCs and stimulate bone mineralisation in zebrafish [187], therefore demonstrating the potential for SB regeneration. Finally, Chen and colleagues [101] showed that berberine (Figure 3e), a plant alkaloid, has osteoinductive properties and is capable of promoting osteochondral regeneration in vivo, combined with an interpenetrating network scaffold of sodium hyaluronate and sodium alginate.

A significant limitation of small molecule therapy is its lower target specificity compared to protein agents. This may hinder the determination of the molecular mechanisms through which small molecules exert their effects, due to the abundance of potential target effectors, but, more importantly, it may result in deleterious side effects due to unspecific action in untargeted tissues and cell populations [177]. As with any other therapeutic candidate, extensive safety screenings need to be performed to address any small molecule interactions with off-target tissues and verify whether toxic effects arise from this low specificity.

**Table 1:** Advantages and disadvantages of the use of growth factors, gene delivery, and small molecules as biochemical stimuli in OC therapy.

Type of Biochemical Stimulus	Advantages	Disadvantages	Examples	References
**Growth factor/chemokine**	Specific action and fewer off-target interactions; Efficient mimicking of physiological signalling cascades	Protein instability in non-native conditions; Short half-life times after administration; High cost	bFGF	[150,154]
			BMPs	[145,146,147,148,155,157,158,159]
			IGF-1	[149,158]
			TGF-β1	[141,142,153,154,155,156]
			TGF-β3	[143,144,157,159]
			SDF-1α	[151,153]
**Protein-coding gene**	Specific, long-lasting action and higher stability of DNA compared to protein agents	Immunorecognition of viral vectors; Low efficiency of non-viral vectors; Difficulty in achieving optimal concentrations of target proteins	BMP-2	[172,173,174]
			TGF-β3	[172,173,174]
			Sox9	[172,175]
			IL-1Ra	[174]
**Small molecule**	Simple administration; Easy high-throughput screening with low cost; Dose-dependent effects allow for a fine-tuning of the therapeutic concentrations	Off-target systemic interactions may result in adverse side effects	Y27632	[151]
			Dexamethasone	[188,189]
			Alendronate	[183,184]
			Berberine	[101]
			KGN	[179,180,181,182,183]
			BNTA	[186]
			DIPQUO	[187]

Abbreviations: bFGF, basic fibroblast growth factor; BMPs, bone morphogenetic proteins; BNTA, *N*-[2-bromo-4-(phenylsulfonyl)-3-thienyl]-2-chlorobenzamide; DIPQUO, 6,8-dimethyl-3-(4-phenyl-1*H*-imidazol-5-yl)quinolin-2(1*H*)-one; IGF, insulin-like growth factor; IL-1Ra, interleukin 1 receptor antagonist; KGN, kartogenin; SDF, stromal cell-derived factor; TGF, transforming growth factor.

#### 3.1.3. Cells

**Figure 4:**
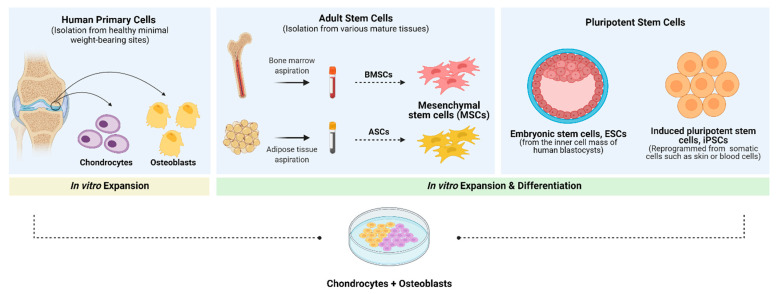
Cell sources explored in the context of cartilage and bone tissue engineering. In contrast to primary cells, which can be derived from healthy load-bearing joints and exhibit the desired osteogenic and chondrogenic phenotypes, adult and pluripotent stem cells need to be expanded, differentiated and/or genetically manipulated to obtain the appropriate cell type. Adult stem/progenitor cells can be isolated from mature tissues such as the bone marrow (via percutaneous bone marrow aspiration) or adipose tissue (via liposuction) and will give rise to bone marrow-derived mesenchymal stem cells (BMSCs) and adipose-derived stem cells (ASCs), respectively. Embryonic stem cells (ESCs) are isolated from the inner cell mass of human blastocysts and induced pluripotent stem cells (iPSCs) can be reprogrammed from human somatic cells such as skin or blood cells, giving rise to chondrogenic and osteogenic cell populations. Created with BioRender.com (accessed on 2 June 2021).

**Figure 5:**
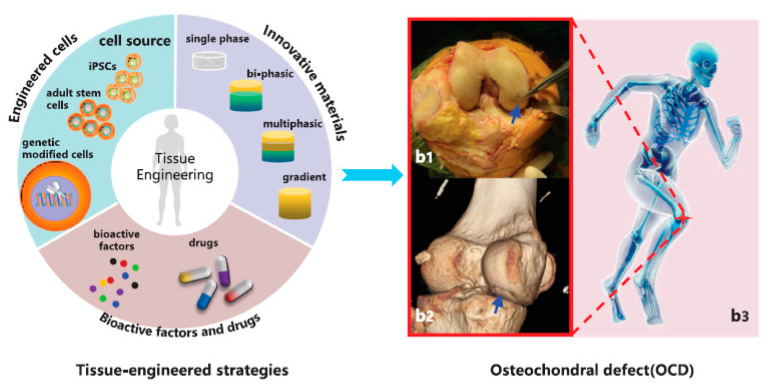
The building blocks of an OC tissue-engineered construct. OCTE strategies usually resort to the combination of innovative biomaterials, cells (e.g., primary or stem cells), and physiologically relevant bioactive molecules or drugs, aiming to recapitulate the biological, physical, and functional features of the native OC unit. Such biomimicking constructs can then be implanted into a damaged OC region, where they will assist tissue repair, promote regenerative responses, and facilitate the functional recovery of the joint. Reprinted from [243] with permission from Wiley Online Library. Copyright © 2020, John Wiley & Sons, Inc.

Although biomaterial scaffolds and biochemical stimuli are crucial components of the TE triad, by providing the 3D supportive environment and chemical cues required for neotissue development, cells are the machinery behind tissue formation. Their presence is especially important when reconstructing tissues with low endogenous regenerative potential, such as cartilage. Thus, the design of biomimetic OC tissues calls for the selection of appropriate osseous and cartilaginous cell sources that meet specific criteria [38,92]. Besides having widespread availability and simple in vitro manipulation, an ideal cell source should be able to produce a matrix resembling that of the native tissue, while providing no immunogenic and disease transmission risk. Moreover, it should also offer the possibility for off-the-shelf access at a low cost, to ensure broad applicability in a clinical setting [190]. Several potential cell sources (Table 2 and Figure 4) have already been described for use in bone [191] and cartilage [192] tissue regeneration. These could be applied in OCTE strategies, as schematically illustrated in Figure 5; however, the selection of a suitable cell source that satisfies the needs of osseous and chondral tissues, as well as of the cartilage-to-bone interface, is still an ongoing issue [38,193,194]. Some strategies focus on using a single cell source with chondrogenic and osteogenic differentiation capacity, while others use multiple cell sources (either primary and/or stem cell-derived) to mimic the bone and cartilage components of the OC unit [38,193].

Despite the wide use of autologous primary chondrocytes and osteoblasts in ACI and MACI-based methodologies, these cells are still associated with several issues that limit their application: (i) cell scarcity, as well as donor-site morbidity and risk of infection upon harvesting; (ii) low proliferation potential during in vitro expansion and high risk of de-differentiation, loss of function or senescence; and (iii) poor characterisation of cell state [38,89]. Notably, these problems become even more relevant when considering large OC defects, in which a greater tissue volume needs to be regenerated. The use of allogeneic or xenogeneic primary cells, which could potentially reduce the problem of availability and morbidity, is linked to potential immunogenicity and disease transmission [195].

As a means to overcome the hurdles associated with primary cells, considerable attention has been given to the use of alternative cell sources, such as stem cells. Among these, human embryonic stem cells (hESCs), adult MSC and, more recently, induced pluripotent stem cells (iPSCs) can be highlighted mostly due to their wide availability, pluri- or multipotency, in vitro proliferation capacity and the ability to differentiate into both osteogenic and chondrogenic cell lineages [193]. To date, both in vitro and in vivo studies have shown the chondrogenic [196,197] and osteogenic [198,199] differentiation ability of hESCs, and expansion protocols have been developed so that hESC-derived cells can be applied in OCTE strategies. Nonetheless, their clinical application is still associated with several constraints, including ethical issues, immunological incompatibilities (given their allogeneic nature), tumorigenic potential in vivo, genomic instability and insufficient understanding of and control over hESC differentiation, which often leads to heterogeneous differentiation [200].

MSCs, also known as mesenchymal progenitor cells, are multipotent undifferentiated cells that can be isolated from several human tissues including the bone marrow, adipose tissue, synovium, periosteum, skeletal muscle, and skin, among others [201]. These cells have been extensively investigated in the context of OC tissue regeneration due to their potential to undergo chondrogenesis and osteogenesis and the fact they can overcome many of the limitations associated with hESCs and primary cells [202]. Autologous MSCs exhibit high immunocompatibility and great proliferative capacity, which means they can, in principle, be indefinitely expanded in vitro without losing their native phenotype [193,203]. Usually, the differentiation potential of MSCs is determined using in vitro models, in which aggregates of MSCs are differentiated by supplementing the culture medium with either osteogenic factors (e.g., dexamethasone, ascorbic acid, β-glycerophosphate) or chondrogenic factors (e.g., dexamethasone, TGF-β, IGFs, BMPs, FGFs) [204]. Nevertheless, mechanical stimuli have also been shown to promote osteogenic and chondrogenic differentiation of MSCs in vitro by the application of tensile strains [205] or hydrostatic pressure and cyclic compression [206], respectively.

Among adult MSCs, BMSCs and ASCs are the most investigated in the context of OCTE. They have already been employed alone (in scaffold-free approaches) and combined with 3D matrices for improved structural support and better integration with the host tissue. Although in vitro studies have revealed that BMSCs have greater chondrogenic [207] and osteogenic [208] potential than ASCs, the latter still attract attention because of their abundance, easy accessibility and the fact they seem to have better long-term genetic stability in culture compared to BMSCs [35]. Moreover, ASCs can be isolated from subcutaneous adipose tissue using minimally invasive methods, thus circumventing donor site morbidity and patient pain, while providing high numbers of cells [35]. In contrast, the isolation of BMSCs from percutaneous bone marrow aspirates usually gives rise to relatively small cell yields that vary between patients; the BMSCs further have a donor age-dependent proliferation potential which declines with age [209,210]. This implies that BMSCs need to be greatly expanded to achieve therapeutic relevance and that their application in an autologous therapeutic strategy might be restricted to younger patients [193,211].

Several studies using BMSCs alone or combined with 3D biomimetic matrices have shown promising results regarding the formation of native-like AC and SB tissues and the overall regeneration of the OC unit upon damage. Jin et al. used osteogenic and chondrogenic pre-differentiated BMSC sheets cultured onto poly-l-lactic acid (PLLA)/gelatine fibrous meshes to obtain a 3D multi-layered gradient scaffold that tried to mimic the hierarchical complexity of the OC interface. In this work, cell/mesh complexes were built layer-by-layer to simulate the cartilage-to-bone transition and then implanted into a rabbit knee defect model for up to 24-weeks. The resultant construct was able to promote OC tissue regeneration and proved that it was possible to construct an intermediate calcified cartilage zone by pre-differentiating BMSC sheets in a chondrogenic/osteogenic inductive medium [212]. Additional in vivo studies by Yang et al. revealed that OC biphasic matrices carrying BMSC-derived chondrocytes and osteoblasts mixed with the corresponding cartilage and bone scaffold layer could promote an almost complete repair of full-thickness AC defects. Indeed, the alginate/gelatine/HA composite scaffolds were firmly integrated with the surrounding tissues and there was evidence of a tidemark, 6 months after implantation in a weight-bearing area of a rabbit knee joint [213].

Likewise, various studies using ASCs have also been reported for OC tissue regeneration. For instance, Moses et al. have used micro-extrusion bioprinting of primed ASC-laden hydrogels to develop silk-based cartilage and bone bioinks capable of replicating the complex OC structure. Besides facilitating the spatial maturation and differentiation of encapsulated stem cells towards osteogenic and chondrogenic lineages, the silk-based bioinks enabled the formation of an undulating demarcation region at the interface of chondral and bone phases. Interestingly, the incorporation of strontium doped nano-apatites as ceramic additives in bone bioink provided osteoinduction and osteocyte maturation of the encapsulated stem cells while supporting SB regeneration and the downregulation of osteoclast activity by aiding endothelial cell survival (proangiogenic effect) [214].

ASCs are isolated from a stromal vascular fraction that also comprises angiogenic cell populations such as endothelial cells, endothelial progenitor cells, and pericytes [193,215]. When considering the design of large-scale OC constructs, the diffusion of oxygen and nutrients becomes a crucial issue that can be attenuated with vascularisation. Hence, endothelial cells could be applied together with ASCs as a strategy to develop functional vasculature in such engineered grafts and subsequently create the possibility to obtain patient-specific cells for a complete OCTE therapeutic approach [193].

Other MSC sources, including synovial tissue and periosteum-derived mesenchymal stem cells (SMSCs and PMSCs, respectively), have also shown potential for osteogenic and chondrogenic differentiation; however, for both cell types, it is still unclear what the long-term outcomes of their application are, in terms of safety and durability. Additionally, further elucidation of their action mechanisms and interindividual heterogeneity, as well as the characterisation of their cellular marker expression profiles are required for future clinical application [216,217]. More recently, MSCs from perinatal tissues such as the umbilical cord (UCMSCs) and the amniotic membrane and fluid (AFSCs) have also been introduced to cartilage and bone TE [173,218,219]. Besides their easy accessibility and high cell yields upon isolation, these cells are thought to retain some primitive features of ESCs and have shown to have broader multipotency than adult MSCs while exhibiting low tumorigenic risk due to their immune-privileged nature. Unfortunately, given their recent introduction in the field, there is still no gold standard methodology for the isolation, purification, and amplification of foetal tissue-derived cells. Moreover, ethical considerations linked to their use have restricted their application [218].

Despite the advantages of MSCs, their use is still associated with challenges and possible risks that require careful assessment before moving forward towards MSC-based clinical applications, including long-term culture anomalies (such as lower proliferation and differentiation potential, lower telomerase activity and morphological changes), heterogeneous differentiation capacity, which hinders the development of standardised protocols for target differentiation, and pro-tumorigenic potential [203,220,221]. Therefore, great attention has been given to iPSCs, which can be obtained by reprogramming terminally differentiated somatic cells through the exogenous expression of pluripotency-associated factors. These cells avoid the ethical issues of ESCs and provide the possibility to generate patient-specific pluripotent stem cells. This way, they overcome the problems related to immunocompatibility and disease transmission, while exhibiting almost unlimited proliferative capacity, which means high cell yields can be obtained for the development of off-the-shelf and personalised therapies—the ultimate goal of TE strategies [193,222]. As a result, since their first introduction by Takahashi and Yamanaka [223], several iPSC-based chondrogenic and osteogenic differentiation protocols have been proposed, as reviewed in recent work by [224,225] and [226,227], respectively.

Given the rapid development of iPSC technology and the fact it is easy to generate iPSCs from skin fibroblasts [228] or blood cells [229], numerous cartilage [225] and bone [230] TE strategies have been proposed and their OC regenerative potential has been evaluated in vitro and in vivo [231,232]. For instance, Nguyen et al. printed human induced pluripotent stem cells (hiPSCs) and chondrocytes embedded in two distinctive nano-fibrillated cellulose (NFC) compositions: alginate (NFC/A) or hyaluronic acid (NFC/HA), employed as cartilage mimics. When cultured within 3D-bioprinted NFC/HA constructs, hiPSCs showed low proliferation and exhibited phenotypic changes indicative of a non-pluripotent state (including spherical morphology); however, for 3D-bioprinted NFC/A constructs (with a 60/40, dry weight % relation), cell pluripotency was initially maintained and a hyaline-like cartilage tissue with collagen type II expression and lacking tumorigenic octamer-binding transcription factor 4 (Oct4) expression was observed, after 5 weeks in culture. This decrease in Oct4 expression is relevant when considering a future clinical implementation since it indicates the reduction of pluripotency, which is usually associated with an elevated risk of tumour formation. Additionally, this work demonstrated that hiPSCs could be directed to a chondrogenic lineage when co-cultured with irradiated chondrocytes and bioprinted into NFC/A-based bioinks [233]. In line with this work, Xu et al. have shown that implanting PLGA scaffolds containing iPSC-derived MSCs into full-thickness defects in a rabbit model can promote the development of cartilage-like tissue, without any teratoma formation, 6-weeks post-implantation [234].

Regardless of the promising results obtained with iPSCs, one major obstacle in their clinical application is the risk of teratoma formation. Although teratoma development after injection into immune-compromised animals is usually the gold standard method for evaluating cell pluripotency in vitro, the risk of producing tumour-like structures in vivo, even if benign, creates important concerns regarding the safety of iPSCs used for transplantation [235]. Different strategies have been proposed to overcome this problem and ensure that a stable cell lineage commitment is maintained in vivo, namely the direct use of terminally differentiated iPSC-derived cells instead of undifferentiated iPSCs, and the direct conversion of somatic cells to chondrocytes and osteoblasts. Regarding the first strategy, iPSCs must be expanded before being differentiated into tissue-specific cells, since many iPSC-derived cells (including iPSC-derived chondrocytes) can undergo dedifferentiation when subjected to in vitro expansion. Further, in vitro differentiation does not guarantee stable lineage commitment and phenotypes in vivo. There are unpredictable and unknown cell-cell and cell-ECM interactions in situ, and therefore teratoma formation can still occur upon transplantation [225]. The second strategy refers to the direct conversion of somatic cells (such as dermal fibroblasts or blood cells) into the desired phenotypes, by circumventing the pluripotent stem cell state. Studies by Tsumaki and colleagues have hypothesised that the misexpression of some reprogramming factors and chondrogenic factors in dermal fibroblasts might result in their conversion to hyaline chondrogenic cells (iChon cells) by erasing important fibroblastic features. In fact, these authors identified specific transcription factors that could promote such conversion and avoid the need for an intermediate iPSC and could obtain homogeneous cartilage-like tissues from both mouse and human iChon cells upon grafting in nude mice [236,237,238]. More recently, Wang et al. have expanded this potential by developing a reprogramming strategy that allows the efficient derivation of osteo-chondrogenic cells [239], which may be promising for both cartilage and bone cell-based therapies.

Another important issue related to the use of iPSCs is immunocompatibility. While patient-specific iPSCs (with autologous origin) do not induce immune responses, allogeneic iPSCs might exhibit tolerance issues. This problem, together with the high costs associated with iPSC preparation under current good manufacturing practice (cGMP) guidelines, might be overcome by generating a bank of allogeneic clinical iPSC lines [240,241]. Such a library could be prepared from homozygous donors with common human leukocyte antigen (HLA) types and could provide an off-the-shelf possibility for obtaining iPSCs at any needed moment. Chondrocytes and osteogenic cells induced from iPSCs with an HLA type that matches the patient’s HLA types could be selected from the iPSC library and used for application in TE (scaffold-based approaches) or directly used for transplantation (scaffold-free approaches) [242]. This innovation could also facilitate iPSC accessibility and allow wider clinical application.

##### Mimicking the In Vivo Physiological Environment: Dynamic Culture Conditions

Regardless of the cell source chosen, the design of biomimetic OC constructs largely depends on our ability to replicate the in vivo environment of the OC unit, which requires keeping an adequate supply of nutrients, oxygen and other biochemical factors during cell culture in vitro. Proper mass transfer of nutrients and metabolic waste products is still a massive challenge when engineering cartilage and bone tissues of clinically relevant sizes. Especially when cultured under static conditions, 3D tissue-engineered constructs typically exhibit a heterogeneous composition and structure, with a hypoxic necrotic central region and localised tissue growth in the construct periphery [244,245,246]. This becomes critical in OCTE since OC defects usually are many millimetres in size, and it is difficult to provide sufficient fresh medium circulation through engineering constructs larger than hundreds of micrometres. Additionally, the static culture of cell-seeded 3D scaffolds cannot yet meet the multicellular complexity and gradients found in OC tissues and requires discrete instead of continuous medium exchange, which further limits the applicability of these systems [244].

To achieve more physiologically relevant OC tissue substitutes, both bioreactors and microfluidic devices have been investigated. In these systems, environmental conditions such as temperature, pH, levels of oxygen, nutrients, metabolites, and other molecules can be tightly controlled, and physiologically relevant physical signals (e.g., shear, pressure, compression, stretch) can be provided while ensuring a spatially uniform distribution of cells [247]. However, even when creating conditions that better replicate the in vivo OC environment, there are still challenges when designing functional engineered OC matrices. Given the distinct biochemical and biomechanical features of cartilage and bone tissues, it is important to ensure that each section of the scaffold exhibits site-specific properties that can induce and maintain osteogenesis and chondrogenesis in the corresponding region. This includes not only the application of different culture media but also the use of precise mechanical stimuli. For instance, while several studies have reported that dynamic laminar flow patterns with concomitant compression can enhance cartilage ECM stimulation, for bone, culture medium perfusion has been shown to affect the geometry, distribution and orientation of the forming bone-like trabeculae, and to provide shear-stress to stimulate neobone formation [190,248].

In simple terms, both bioreactors and microfluidic systems consist of cell culture environments confined in a vessel-, flask- or channel-like reservoir, connected to inlet and outlet stations for continuous flow of nutrients and oxygen. Bioreactors allow the development of cell-material constructs under a controlled environment, often using mechanical methods to direct biological processes (i.e., cell differentiation and ECM formation) [190,244]. Microfluidic devices allow spatial control over fluids in micrometre-sized channels and represent a useful technique to reduce either the time or costs of cell culture processes and diagnostic systems. As a result of the miniaturised nature of microfluidic devices compared to bioreactors, and the fact that tissue-engineered OC constructs might need to be scaled up to the millimetre range, more emphasis will be given to the studies focusing on bioreactor systems. Among the various types of bioreactors, spinner flasks, rotating vessels, perfusion systems and compression bioreactors stand out in OCTE [244].

Spinner flask bioreactors are some of the simplest and most frequently used models. In these bioreactors, cell-based scaffolds are suspended from needles connected to the top cover of the container. There is a magnetic stirrer bar at the bottom that mixes the medium across the scaffold(s), as well as inlets that allow for gas exchange. Unfortunately, due to the use of a magnetic stirrer, spinner flasks are associated with high shear stresses that have been shown to harm neotissue formation [244,249]. In contrast, the rotating wall vessel bioreactor system consists of two cylindrical containers, within which lies a ring-shaped space containing the freely suspended tissue-engineered constructs [250]. As the cylindrical vessel rotates horizontally around its axis, its contents accelerate until the whole fluid mass is rotating at the same rate as the vessel wall, simulating microgravity conditions and inducing dynamic laminar flow conditions [251]. In this environment, the damaging effects of turbulence and shear stress are minimised compared to the spinner flask model [244,250].

In the case of perfusion bioreactors, there is a pump system that can perfuse media through tissue-engineered constructs (accommodated inside chambers or columns) in a controllable and continuous manner, which ensures improved fluid transport capacity [244,249]. In fact, in these systems, culture media can flow through the interconnected pores of a solid construct, providing a uniform cell distribution and high seeding efficiency throughout the full engineered scaffolds. Finally, compressive bioreactor systems usually consist of a motor, a system providing linear motion, a controlling mechanism providing displacement regimes, and a compression chamber that applies static or dynamic compressive loads directly to the cell/scaffold constructs. In these models, the compressive force is transferred to the construct by flat plates that distribute the load evenly, ensuring uniform stimulation of the grafts [252].

**Figure 6:**
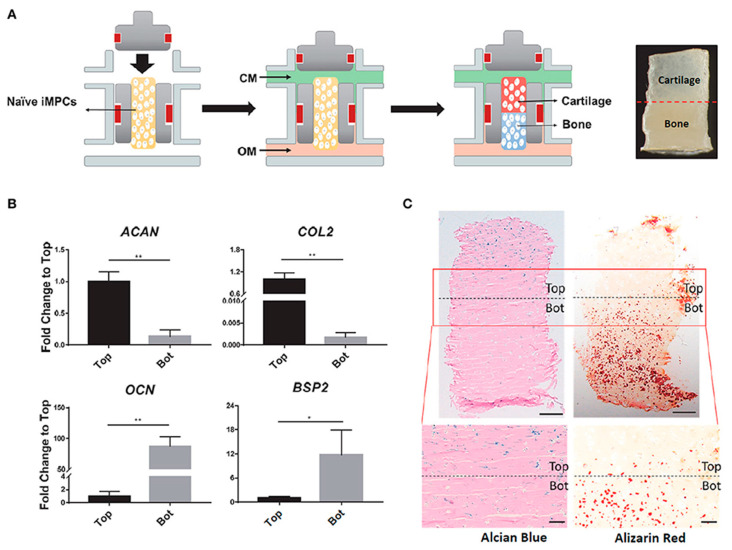
Potential of dual-flow perfusion bioreactors for the development of hiPSC-derived OC tissue. (**A**) Schematic representation of the OC tissue construct containing cartilaginous and osseous layers, as well as of the dual-flow bioreactor system where the constructs were placed and perfused with optimised chondrogenic (CM) and osteogenic (OM) media through the top and bottom flow, respectively, to promote the formation of the biphasic tissue. (**B**) Characterisation of the engineered OC construct in terms of the expression levels of chondrogenic (aggrecan—ACAN, collagen type 2—COL2) and osteogenic (osteocalcin—OCN, bone sialoprotein 2—BSP2) markers, in the top (Top) and bottom (Bot) sections of the construct, 28-days after differentiation; * *p* < 0.05; ** *p* < 0.01. (**C**) Histological examination of the biphasic OC tissues as regards the deposition of the tissue-specific matrix; Alcian Blue positive staining is restricted to the top of the construct (cartilage), whereas Alizarin red positive staining is limited to the bottom part (bone). Scale bar = 500 μm (**top panel**); Scale bar = 200 μm (**bottom panel**). Adapted from [261] with permission from Frontiers. Copyright © 2007–2021 Frontiers Media SA.

Each model has important strengths and limitations, either in terms of system complexity and operation, as well as regarding cellular outcomes [244]. For instance, Song et al. showed that the dynamic culture of OC biphasic composites comprising cell-hydrogel and cell-cancellous bone constructs in a spinner flask contributed to the formation of an interface region, cell infiltration and distribution in the OC composites. This, combined with mechanical stimulation, promoted osteogenic and chondrogenic differentiation of hASCs, as suggested by the increased expression of ALP and GAG in the bone and cartilage replacement materials, respectively [253]. Although a better performance was shown for OC composites cultured under dynamic conditions, spinner flasks are often associated with turbulence and shear stress that could lead to serious damaging effects. In contrast, perfusion-based bioreactors can provide a more homogenous mixing of the medium and allow an improved environmental control and physical stimulation of the cells in large constructs, thus overcoming the difficulties of simpler models [254]. Numerous studies reporting the use of perfusion-based bioreactors in cartilage [255,256,257], bone [254,258] and OCTE [259,260,261] can be found in the literature. In fact, Lin and colleagues have developed microphysiological OC tissue chips derived from human iPSCs using a dual-flow bioreactor, with both chondrogenic and osteogenic media streams [261]. In this study, iPSCs were first induced into mesenchymal progenitor cells (iMPCs) and then differentiated into chondrogenic and osteogenic lineages after encapsulation in photo-crosslinked gelatine scaffolds. After 28 days of culture under differentiation conditions, OC tissue chips were successfully formed, and chondral and osseous phenotypes were validated by specific gene expression and matrix deposition (Figure 6). The neobone tissue formed could promote chondrogenesis and suppress chondrocyte terminal differentiation in the chondral tissue, which suggests that there is functional crosstalk between cartilage and bone components in the OC tissue chip. Furthermore, this chip represents a high-throughput platform applicable for modelling OC-related diseases such as OA [261]. Alas, perfusion-based devices still lack key components, including the possibility for mechanical stimulation of the tissue-engineered constructs during dynamic culture, which can be attained by compressive or hydrostatic systems.

Although advantageous in many ways, the use of compressive and hydrostatic pressure bioreactors still needs a further understanding of specific mechanical loading and regimes of application (i.e., magnitude, frequency, continuous or intermittent use and time of application) [246]. Indeed, to provide adequate stimuli to OC-engineered constructs, complex systems involving the application of both dynamic compression and medium perfusion need to be developed. Additionally, engineered tissues at different stages of maturation might require distinct mechanical conditioning due to the increasing ECM accumulation and developing structural organisation [262]. This means that both a spatial and time-dependent application of loading stimuli is required to ensure the formation of fully functional neotissue capable of integration into the host, for which dual-chamber bioreactors might be valuable. It is also important to note that these bioreactor systems are very complex for use in large-scale production and/or high throughput uses, which has hindered their wider application. Therefore, given the complex demands of OCTE, combinations of distinct bioreactors types have been explored to better mimic the OC physiological environment in vitro, including compression bioreactors combined with perfusion [263,264,265,266].

Recently, Lovecchio et al. developed a standalone perfusion/compression bioreactor system specifically for inducing osteogenic commitment of BMSCs seeded on 3D chitosan-graphene templates. Comparing to static culture conditions, the application of perfusion and compression stimuli for one week led to a considerable increase in cell number and enhanced ECM mineralisation [263]. Based on these studies, it is possible to conceive the design of more complex systems for application in OC composite constructs. Other relevant bioreactor systems being explored include bioreactors mimicking the multi-axial motion of an articulating joint [267] or perfusion seeding systems [268,269]. Direct perfusion of a cell suspension through a 3D scaffold has been shown to be an effective method to enhance the distribution of cells throughout an entire scaffold and to contribute to the formation of more homogeneously distributed tissue [270]. Indeed, this seeding method should overcome the operator-dependent limitations associated with cell static loading (i.e., micropipetting) [245]. Moreover, these systems can be integrated into a more complex bioreactor setting in which the cells are first perfused through the scaffold and then kept under specific dynamic culture within the engineered construct.

### 3.2. Building Block Assembly: Scaffold Fabrication and Characterisation

Because OC regeneration relies on multi-factorial approaches, the development of methods for OC scaffold manufacturing that allows for fine-tuning of the architectural, biomechanical, and biochemical properties of the tissue-engineered constructs is an ultimate goal. Accordingly, electrohydrodynamic techniques, such as electrospinning, and additive manufacturing (AM) have presented themselves as ideal candidates for OC scaffold production and optimisation. While sharing some technical similarities, these two methods offer different kinds of control over scaffold morphology, mechanical behaviour, and biological response.

#### 3.2.1. Electrospinning

**Figure 7:**
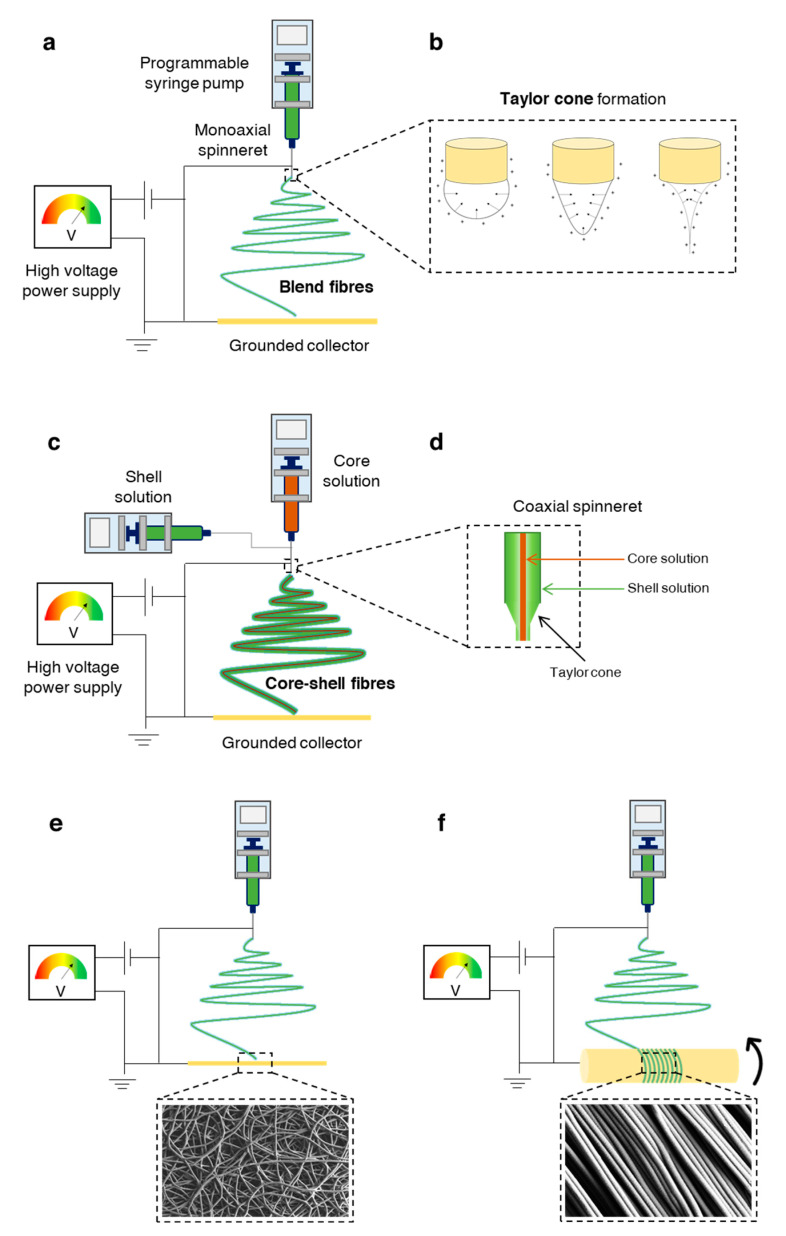
(**a**) Basic electrospinning setup. A programmable syringe pump is used to infuse a polymer solution, emulsion, or melt through an electrically conductive spinneret (e.g., a metallic needle), to which a high voltage (usually 5–20 kV) is applied. The accumulation of electrical charges in the polymer droplets at the tip of the spinneret leads to repulsive forces that eventually result in the formation of a cone shape, the Taylor cone (**b**), which is followed by the ejection of the liquid. While the majority of electrospinning studies report the use of a positive voltage at the nozzle, negative voltages can be applied instead (see ref. [284]). The jet is then accelerated towards a grounded or oppositely charged collector, the solvent is evaporated, and dry fibres are deposited. When a monoaxial (single-channel) spinneret is used, blend fibres are formed. Core-shell fibres can be produced using coaxial electrospinning (**c**), with the help of a coaxial spinneret (**d**) in which an inner solution (core), usually containing an active pharmaceutical ingredient, is enveloped by an outer solution (shell). The organisation of the deposited fibres can be controlled using different collector architectures: a flat collector (**e**) will generate randomly deposited fibres, while a cylindrical mandrel (**f**) rotating at high speeds will result in highly aligned fibres. The Taylor cone schematic representation in (**b**) was adapted from [285] with permission from Elsevier. Copyright © 2020, Elsevier B.V. The scanning electron microscopy (SEM) images in (**e**,**f**) were adapted from [275] with permission from Elsevier. Copyright © 2015, Elsevier Inc.

Electrospinning is an electrohydrodynamic technique based on the extrusion of a polymer solution, emulsion or melt through a spinneret under the application of a strong electric field, resulting in fibre production and deposition on an appropriate collector. The basic experimental set-up (Figure 7a,b) is composed of one or several syringe pumps, a conductive nozzle (spinneret), a high-voltage power supply, and a grounded collector. A polymer solution is infused by the syringe pump through the nozzle and accelerated, using a high potential difference (kV), towards the collector; during this process, the solvent is evaporated and a dry fibrous mesh is deposited onto the surface [271]. Complex geometries, like core-shell fibres, can be attained using variations of this method, such as coaxial or multiaxial electrospinning (Figure 7c,d). Multicomponent fibre composition can equally be attained by side-by-side electrospinning of two polymer solutions arranged in a parallel and adjacent manner [272,273]. Randomly arranged or aligned fibres can also be obtained by varying the type of collector used: a flat metal plate will give rise to random fibre deposition (Figure 7e) and a cylindrical mandrel rotating at high speed can produce fibre alignment (Figure 7f) [274,275]. Similarly, morphological properties like fibre diameter can be adjusted by tuning solution (polymer concentration/molecular weight and solvents used, which affect solution viscosity and conductivity), processing (flow rate, nozzle-to-collector distance, voltage), and environmental parameters (temperature, humidity) [276].

**Figure 8:**
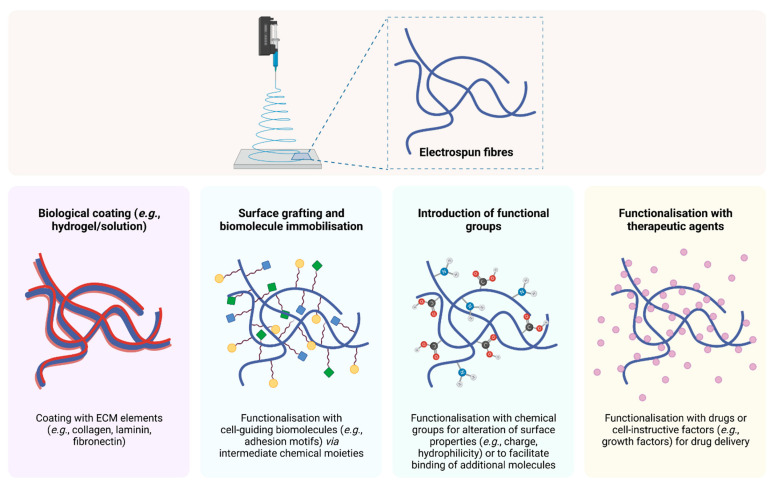
Examples of post-fabrication surface modifications of electrospun fibres. These can be obtained by physical or chemical modification techniques, such as plasma treatment or wet chemistry. Created with BioRender.com (accessed on 9 June 2021).

An extensive list of natural (e.g., chitosan, collagen, silk fibroin, gelatine) and synthetic (PCL, PLGA, PLA, poly(ethylene oxide) (PEO), PEG, etc.) polymers is compatible with electrospinning, allowing the generation of fibrous meshes with variable chemical composition and associated properties. Importantly, this technique generates fibre architectures that closely resemble those of the native ECM, not only with adjustable fibre diameter and orientation but also with interconnected porosity and high surface area-to-volume ratios [277]. In addition, fibres produced by electrospinning can be subjected to post-processing surface modifications (Figure 8) to further adjust the physicochemical properties and biological response of the scaffold. Common surface modification techniques include plasma treatment, wet chemistry, and physical or chemical functionalisation with biological ligands or drugs for controlled delivery [278]. This versatility of post-fabrication adjustments allows the modulation of cellular adhesion, proliferation, and differentiation, representing an important route through which biological responses can be tuned to affect tissue repair. Plasma treatment is one of the most frequently used surface modification techniques and it has been shown in multiple studies to enhance osteogenic and chondrogenic differentiation of hMSCs and hiPSCs, as well as encourage cell attachment and proliferation (reviewed in detail in ref. [278]). Nanofibre surface functionalisation with chondroitin sulphate using polydopamine as an intermediate ligand markedly stimulated rabbit chondrocyte and BMSC adhesion, proliferation, and chondrogenic differentiation, resulting in improved in vivo regeneration after implantation into a rabbit chondral defect [279]. Chemical modifications (e.g., amination) also enable alterations to the surface charge of the scaffolds, which has been demonstrated to influence osteogenic differentiation of MSCs [280,281,282]. The fact that electrospinning is a scalable technology with real potential for industrial mass production constitutes another advantage [283].

The wide diversity of structures that can be generated using electrospinning has been demonstrated by several recent studies [286,287,288,289]. Liu et al. [286] developed a multi-layered scaffold where an electrospun artificial calcified cartilage layer was included between the AC and SB phases. The structure of calcified cartilage was emulated by using a stratified fibrous membrane of silk fibroin, chitosan, and hydroxyapatite, organised in such a way that both a chemical (hydroxyapatite) and a physical (porosity) gradient were established between the artificial bone and cartilage layers. PEO was also added to the polymer blend to improve fibre morphology and facilitate the dispersion of the hydroxyapatite nanoparticles within the solution. The intermediate layer was found to be selectively permeable to low molecular weight molecules (glucose, fluorescein isothiocyanate (FITC)-conjugated dextran (DEX) of 4 kDa) but blocking the diffusion of larger compounds (FITC-DEX of 10 and 70 kDa). Moreover, the electrospun membrane seemingly prevented the migration of cells from the bone layer to the cartilage layer and vice-versa, mimicking, to some extent, the properties of native calcified cartilage [286].

In another study, a biodegradable thermosensitive hydrogel was developed by Brunelle and co-workers, where PCL was combined with a copolymer of PEG and poly(N-isopropylacrylamide) (PNIPAAm) to produce composite electrospun scaffolds in a single step method [287]. While PCL provided structural support, the thermosensitivity of PEG-PNIPAAm allowed the loading of the construct with hMSCs at room temperature and subsequent gelation at 37 °C, a characteristic of interest for in vivo and human implantation. Composite hydrogels fabricated with 65% PEG-PNIPAAm in the polymer blend were biocompatible and promoted a homogeneous cell distribution throughout the constructs, with the production of ECM and expression of type II collagen, aggrecan, and Sox9 coding genes [287]. Likewise, a poly(3-hydroxybutyrate-*co*-3-hydroxyvalerate) (PHBV) nanofibre-reinforced chitosan/silk fibroin hydrogel was produced, where the PHBV fibres were generated by wet electrospinning (fibre collection into a liquid medium) [288]. The electrospun fibres in suspension were added to chitosan:silk fibroin (1:1 *v*/*v*) solutions, after which crosslinking by poly(ethylene glycol) diglycidyl ether (PEGDE) allowed the gelation and formation of the composite hydrogel. Constructs with a chitosan/silk fibroin:PEGDE ratio of 1:1 (*w*/*w*) demonstrated an interconnected porous structure and a high-water content (~91% of the scaffold’s wet weight), and reinforcement with PHBV electrospun fibres resulted in an improvement of the hydrogel’s mechanical properties. Moreover, the composites successfully promoted proliferation and GAG production by rat BMSCs [288].

**Figure 9:**
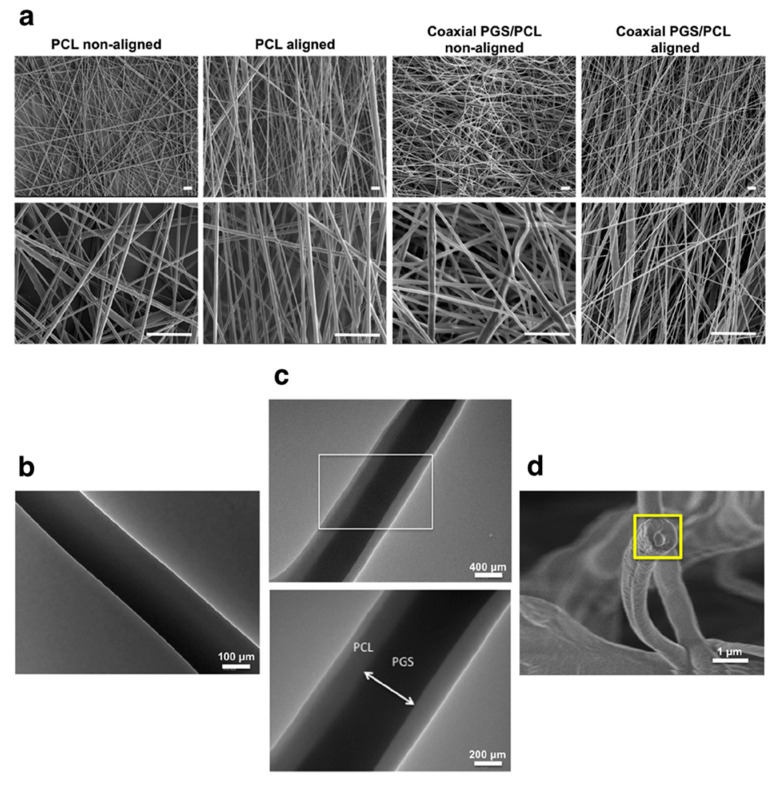
Monolithic and core-shell electrospun fibres. Scanning electron microscopy (SEM) images (**a**) of aligned and non-aligned monolithic PCL and core-shell PGS-PCL fibres at two different magnifications. Scale bars: 5 μm. Transmission electron microscopy (TEM) images of (**b**) PCL monolithic fibres and (**c**) PGS-PCL core-shell fibres. The bottom panel in **c** is a magnification of the area within the white box in the top panel. The core-shell structure of PGS-PCL fibres was also confirmed by SEM imaging of the fibre cross-section (**d**, yellow box). Abbreviations: PCL—poly(ε-caprolactone); PGS—poly(glycerol sebacate). Adapted from [289] with permission from Elsevier. Copyright © 2019, Elsevier B.V.

Coaxial electrospinning was employed to generate poly(glycerol sebacate) (PGS, core)-PCL (shell) nanofibres, in which KGN was encapsulated [289]. Aligned PGS-PCL and PCL fibres were compared to randomly deposited core-shell and PCL monolithic fibres (Figure 9). In accordance with previous studies [274], aligned fibres presented improved mechanical properties when compared to randomly arranged fibres, which led to an enhancement in the elastic moduli of aligned constructs by up to 2.5-fold. KGN was released in a sustained manner from coaxial PGS-PCL fibres for 21 days, whereas release from monoaxial PCL fibres was much faster and associated with a more pronounced burst of release on the first day. Importantly, all scaffolds supported hBMSC attachment and proliferation, as well as ECM production and expression of chondrogenic genes (type II collagen, Sox9, aggrecan, and proteoglycan 4 (PRG4)), particularly KGN-loaded fibres [289].

Another valuable advantage of electrohydrodynamic techniques is how they enable the encapsulation of bioactive elements with distinct chemical structures, including not only small molecules but also DNA [290,291] and proteins [285,292,293]. Cell electrospinning has also been performed, allowing the direct incorporation of live cells during the scaffold manufacturing process [294]. Recently, the incorporation of resveratrol into PLA-gelatine electrospun scaffolds was shown to highly improve the regeneration of a full-thickness osteochondral rat defect, when compared to unloaded scaffolds or empty defects [295]. Histological and immunohistochemical analyses revealed that the defects treated with resveratrol-encapsulating scaffolds displayed greater chondrocyte organisation, ECM deposition, SB formation, and overall lesion recovery. Inorganic compounds, such as hydroxyapatite, can also be encapsulated within electrospun fibres [286]. Zinc oxide (ZnO)-containing PCL electrospun fibres have demonstrated potential for the chondrogenic and osteogenic differentiation of hMSCs at low and high ZnO concentrations, respectively [296]. Additionally, electrospinning can be used for the development of growth factor delivery systems for OC regeneration. IGF-1 encapsulated within PLGA/PCL blend electrospun fibres demonstrated chemotactic activity in vitro, promoting cell migration from a cartilage explant, and stimulated ECM deposition by articular chondrocytes [149].

**Figure 10:**
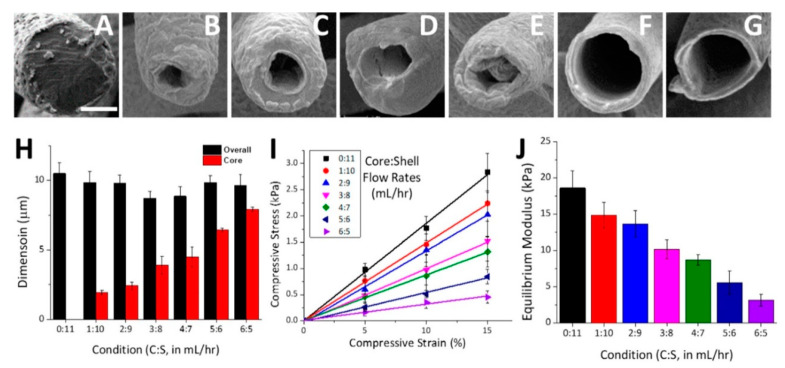
Adjustment of the mechanical properties of electrospun fibres. SEM images of the cross-sections of hollow fibres fabricated by varying the core (PEG):shell (PCL) polymer flow rate ratios (in mL/h) during coaxial electrospinning: (**A**) 0:11, (**B**) 1:10, (**C**) 2:9, (**D**) 3:8, (**E**) 4:7, (**F**) 5:6, and (**G**) 6:5. Scale bar: 5 μm. The PEG core was subsequently removed using PBS. (**H**) Overall and core diameters of the hollow fibres for each core:shell flow rate ratio. (**I**) Representative compressive stress-strain curves from electrospun scaffolds (3 mm thickness) fabricated with the different hollow fibres displayed in (**A**–**G**). (**J**) Equilibrium moduli of electrospun scaffolds demonstrate that the mechanical properties decrease substantially for higher core flow rates (since these result in a larger hollow compartment after PEG leaching). Reprinted with permission from [297]. Copyright © 2019, American Chemical Society.

In addition, electrospinning allows the manufacturing of OC scaffolds with mechanical gradients, in order to better mimic the physiological microenvironment of the joint. Using coaxial electrospinning, Horner and co-workers [297] developed an osteochondral scaffold with depth-dependent strain under dynamic compressive loading conditions. This was accomplished by the manufacturing of a fibrous construct composed of a PCL shell and a PEG sacrificial core. By varying the core:shell component ratios and dissolving the PEG core after fabrication, several layers of monolithic and hollow fibres were continuously deposited to give rise to a monolithic scaffold with gradient mechanical properties (Figure 10). Interestingly, spatial regulation of hMSC differentiation was achieved with this strategy: in high strain regions, the expression of chondrogenic markers, such as aggrecan and type II collagen, was up-regulated; conversely, osteogenic markers (type I collagen, Runx2) were expressed more abundantly in lower strain regions [297]. In a different study, a silk fibroin scaffold with gradient pore dimensions was produced by low-temperature electrospinning, in which fibres were collected onto a cooled surface (−50 to −80 °C) [298]. The authors demonstrated that the scaffold’s pore diameter greatly influenced cell attachment and viability: the ideal condition was that of intermediate pore size, large enough to allow cell migration into the scaffold, but sufficiently small to ensure an appropriate surface area for cell attachment. The influence of pore size and shape on cell migration was further proved by another report, in which the higher porosity and pore interconnectivity of randomly deposited PLGA scaffolds compared to their aligned counterparts allowed for deeper penetration of murine MC3T3-E1 osteoblasts into the fibres [299]. These studies demonstrate that cells are capable of responding not only to chemical stimuli but also to biomechanical cues, therefore reinforcing the importance of having suitable mechanical properties in an OC scaffold.

Potential pitfalls associated with electrospinning include poor control over scaffold architecture, difficulty in producing 3D structures, and usually small pore size that may hinder cell penetration and migration, as well as nutrient circulation and waste removal [298,300]. The process is also highly dependent on environmental parameters such as temperature and humidity, which can be difficult to control [276,301]. Additionally, most electrospinning techniques make use of organic solvents, due to their higher volatility and lower surface tensions facilitating the electrohydrodynamic extrusion of the polymer solution and ensuring efficient solvent evaporation during fibre formation. This raises environmental concerns in terms of the large volumes of organic solvent waste that would potentially be generated with mass production of electrospun fibres; furthermore, the presence of trace amounts of these solvents in medical products may compromise their safety and approval by regulatory entities. Attempts at enhancing electrospun scaffold porosity and obtaining 3D structures include combinations of electrospinning with porogen (e.g., salt) leaching [302], gas foaming [303,304], low-temperature/cryogenic spinning [298,305], specialised collectors [306,307], or the incorporation of sacrificial components that can be easily removed by dissolution after fibre production [308,309]. Si and co-workers have also generated 3D constructs from electrospun fibres by fragmentation of the fibre mats after electrospinning and subsequent homogenisation in water:*tert*-butanol (4:1 *w*/*w*), giving rise to uniform fibre dispersions. Such dispersions were then poured into a mould, frozen in liquid nitrogen, freeze-dried, and crosslinked, resulting in ultralow density, highly porous, but superelastic aerogels [310]. Using a similar post-spinning fibre homogenisation and freeze-drying approach, followed by crosslinking with hyaluronic acid, a biocompatible and superabsorbent 3D gelatin/PLLA scaffold was developed by Chen and colleagues, capable of supporting the growth of rat chondrocytes in vitro and demonstrating in vivo regenerative potential in a rabbit OC defect model [311].

In addition, numerous reports have explored different 3D electrospinning approaches for the generation of 3D scaffolds with improved pore size and overall architecture. Xu et al. [312] made use of a wet electrospinning technique, in which poly(L-lactide-co-ε-caprolactone)/collagen blend fibres were electrospun into a water vortex and subsequently collected onto a rotating mandrel and freeze-dried, forming nanoyarn scaffolds for use in tendon tissue engineering. These scaffolds presented larger pore sizes and increased porosity compared to traditionally electrospun random and aligned scaffolds, which resulted in greater tendon cell penetration, proliferation, and gene expression of tendon-specific markers. A recent study [313] has described the generation of 3D electrospun structures using specialised hydrogel collectors, which can be either removed post-spinning, forming hollow structures (e.g., for blood vessel TE), or maintained, resulting in hydrogel scaffolds lined with electrospun fibres. Importantly, drug/cell-loaded hydrogels can be used as collectors and thus biological delivery systems can be developed using this technique.

Moreover, 3D electrospinning can be performed based on fibre self-assembly. This method relies on the electrostatic polarisation of the deposited fibres, i.e., on the generation of a negative charge on the deposited fibre mats during the electrospinning process, attracting the positively charged polymer jet and working as preferential collection surfaces for the newly formed fibres [314]. Various 3D constructs with improved porosity, a thickness of several centimetres, and complex self-assembled nanostructures, such as honeycomb patterns, have been produced using this technique [315,316,317,318]. For further detail, several recent reviews describe thoroughly the different 3D electrospinning techniques currently in use [314,319,320,321].

In order to avoid the use of organic solvents, alternative electrohydrodynamic techniques such as melt electrospinning may be favoured. Melt electrospinning, as the name implies, relies on the extrusion of polymer melts instead of solutions or emulsions, therefore eliminating the use of solvents in the process [271]. Solid fibre formation is thus carried by cooling of the polymer from the spinneret to the collector, rather than solvent evaporation. Of note, melt electrospinning is associated with higher productivity rates and lower costs than classic solution electrospinning [322]. Nevertheless, because this technique has only recently started being widely explored, the list of materials that can be used is still short, and the high processing temperatures preclude the encapsulation of thermolabile bioactive cues, like proteins [271,322]. In addition, the fibre diameters originated by melt electrospinning are usually larger (>1 μm) than those produced by traditional spinning methods [283]. As discussed in future sections, however, the principles of melt electrospinning may be applied as AM technology, which gave rise to a “hybrid” method called melt electrospinning writing (MEW) [323,324]. In fact, electrohydrodynamic direct writing techniques such as MEW and near-field electrospinning (NFES) may overcome the limitations of both electrospinning and traditional AM, providing the means for the production of 3D scaffolds with tuneable architectures and physicochemical properties. This possibility will be further discussed in future sections.

#### 3.2.2. Additive Manufacturing: 3D and 4D Printing

**Figure 11:**
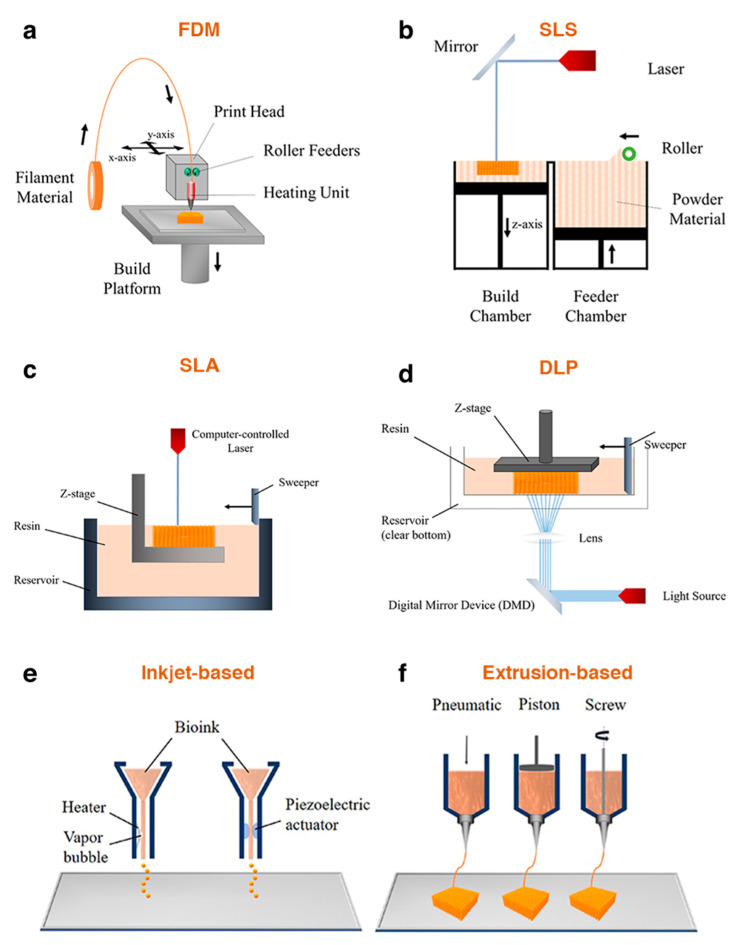
3D printing principles and popular techniques. (**a**) Fused deposition modelling (FDM). A filament of thermoplastic material is continuously fed to a printer head with a heating unit, which melts the material and facilitates its extrusion and layer-by-layer deposition onto a substrate. (**b**) Selective laser sintering (SLS). The projection of a laser in a pre-defined pattern is used to sinter powdered material. Once a layer has been completed, the substrate in the build chamber lowers by a pre-determined distance, according to the computational model used, and new material is fed from the feeder chamber to start a new layer. (**c**) Stereolithography (SLA). A light source (e.g., a laser) is used to solidify liquid and photosensitive material (resin) in the desired pattern. Once a layer is completed, the substrate is lowered vertically and new resin can be polymerised on top. (**d**) Digital light processing (DLP). This is similar to SLA; it uses a digital micro-mirror device (DMD), composed of numerous micro-mirrors that direct and focus the light source to the resin surface according to the designed pattern. (**e**) Inkjet-based 3D printing. An ink or bioink is loaded into a cartridge and can be ejected with the help of heat-generated bubbles or a piezoelectric actuator. (**f**) Extrusion-based 3D printing. The (bio)ink is extruded using pneumatic or mechanic (piston/screw) systems. Adapted from [325] under the terms of the Creative Commons Attribution License (https://creativecommons.org/licenses/by/4.0/). Copyright © 2019, Tamay, et al.

AM, particularly 3D printing, employs layer-by-layer deposition and computer-aided design (CAD) for scaffold production. The most common AM methods are fused deposition modelling (FDM), selective laser sintering (SLS), stereolithography (SLA), extrusion-based 3D printing, and inkjet 3D printing (Figure 11). The main advantage of AM techniques is precise control over scaffold architecture: provided there is a CAD model of an OC defect, which can be generated from high-resolution medical images obtained from magnetic resonance imaging (MRI) or computed tomography (CT), it is possible to generate a construct that perfectly fits this lesion, paving the way for personalised therapy [325]. As such, AM techniques have the immeasurable potential for precision medicine, since they enable mass customisation, generation of constructs with complex geometries, and the use of multiple biomaterials with variable physicochemical properties [326]. Depending on the specific AM methodology, natural [327,328,329] and/or synthetic [135,151] materials, as well as ceramics or metals [330,331], can be used in AM as resins or inks.

**Figure 12:**
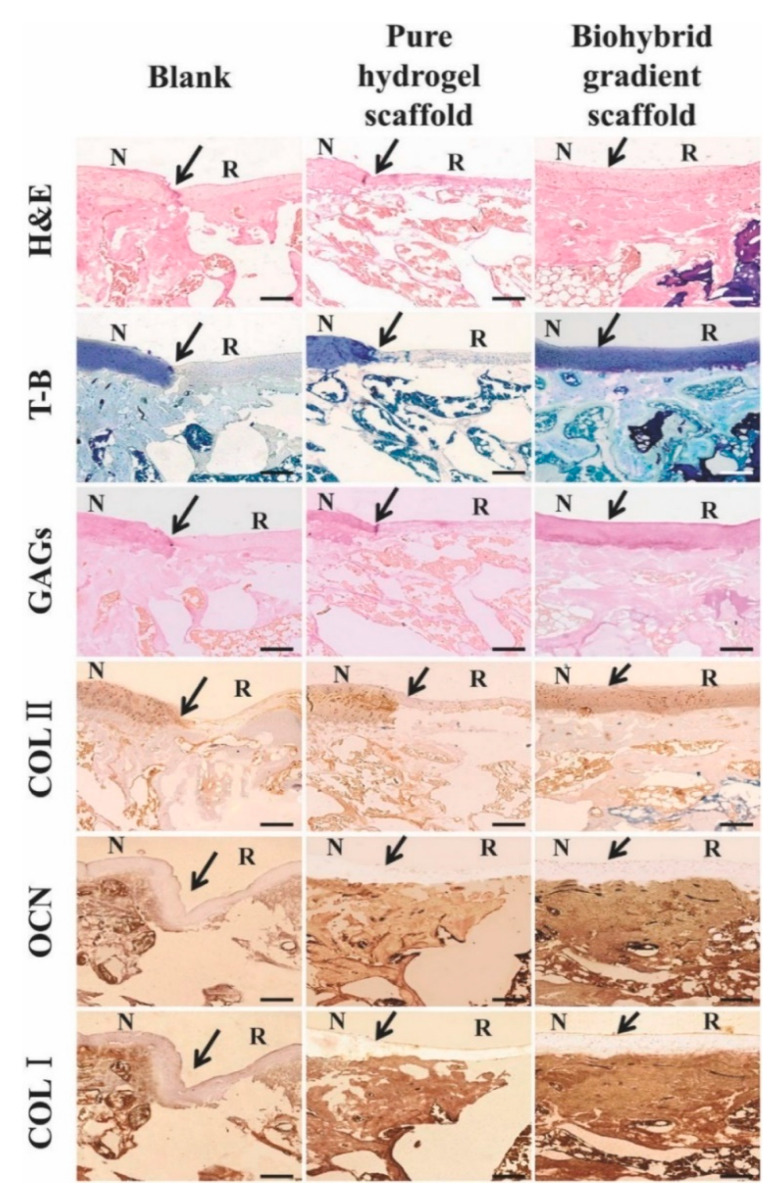
Histological analysis of 3D printed implants 12 weeks after surgery and scaffold implantation in rat OC defects. H&E and toluidine blue (T-B) staining demonstrated that the TGF-β1/β-TCP scaffold (biohybrid gradient scaffold) improved tissue repair and organisation, compared with pure hydrogel scaffolds and no scaffold (blank). The content of GAGs, as assessed by Periodic-Acid Schiff (PAS) staining, and type II collagen, determined by immunohistochemistry, was additionally improved by the biohybrid scaffold. SB formation was also observed, with higher osteocalcin (OCN) and type I collagen contents in the groups treated with the biohybrid scaffold. N—normal cartilage; R—repaired cartilage; black arrows indicate the interface between normal and repaired cartilage. Scale bars: 200 μm. Reprinted with permission from [332]. Copyright © 2018, WILEY-VCH Verlag GmbH and Co. KGaA, Weinheim.

These methods can also allow the incorporation of bioactive molecules into the generated constructs. Poloxamine and PCL blends were loaded with dexamethasone and 3D printed using an FDM technique, giving rise to drug-loaded scaffolds to be used for bone tissue engineering [189]. Dexamethasone-loaded constructs were able to promote MSC growth and higher ALP activity compared to unloaded scaffolds, demonstrating the preservation of the drug functionality throughout the 3D printing process. As another example, a thermal-assisted extrusion printing technique was used to produce biohybrid scaffolds containing TGF-β1 and β-TCP nanoparticles [332]. The bioink consisted of a poly[*N*-acryloyl glycinamide-*co*-*N*-(tris(hydroxymethyl)methyl) acrylamide] copolymer, either unloaded (AC phase) or loaded with thermally stable β-TCP (SB phase). To ensure the preservation of protein integrity, given the high processing temperatures of this technique, TGF-β1 was added to each AC layer after the respective 3D printing process. These hybrid scaffolds had suitable biomechanical properties for OC applications, displaying an elastic behaviour that supported several types of deformation with no visible damage and easy recovery of their initial shape. The preservation of TGF-β1 and β-TCP bioactivity after scaffold manufacturing was first confirmed in vitro, where they respectively enhanced the expression of chondrogenic and osteogenic markers by hBMSCs. Moreover, TGF-β1/β-TCP-loaded scaffolds demonstrated remarkable bioactivity in vivo (Figure 12): their implantation in a full-thickness rat OC defect led to the concomitant regeneration of SB and AC over 12 weeks, with seamless neotissue integration and the formation of a hyaline-like cartilaginous tissue rich in GAGs and type II collagen [332].

**Figure 13:**
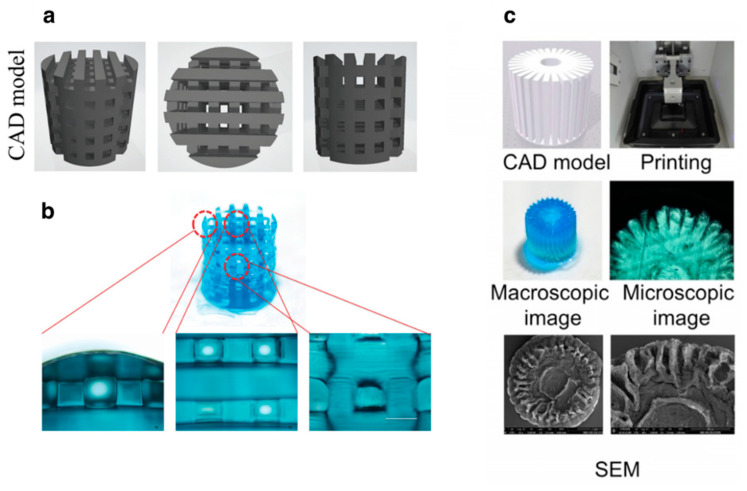
3D-printed scaffolds for OCTE. (**a**) Computer-aided design (CAD) model of a PEGDA/dECM scaffold. (**b**) Macroscopic image of the 3D-printed PEGDA/dECM scaffold. Scale bar: 0.7 mm. Adapted with permission from [329]. Copyright © 2020, SAGE Publications. (**c**) 3D printing process, macroscopic, microscopic, and SEM imaging of the radially-oriented exosome-loaded GelMA/dECM scaffold. Adapted from [333] under the terms of the Creative Commons Attribution-NonCommercial (CC BY-NC) license (https://creativecommons.org/licenses/by-nc/4.0/). Copyright © 2021, Ivyspring International Publisher.

Similarly, promising results were recently achieved by Zhu and co-workers, with the use of a hybrid PEGDA/dECM 3D-printed scaffold (Figure 13a,b) loaded with honokiol, a natural polyphenol with anti-inflammatory action [329]. Upon in vivo testing over eight weeks, comparable SB repair levels were attained for unloaded and honokiol-loaded PEGDA/dECM constructs, revealing that the biomaterials alone can guide bone regeneration. Nevertheless, the presence of honokiol proved crucial for cartilage remodelling and repair, culminating in a well-organised hyaline-like cartilaginous neotissue with stratified chondrocyte disposition close to that of native cartilage and expression of tissue-specific biochemical markers [329]. It should, however, be taken into account that the in vivo studies in both these reports were carried using rat OC defect models, in which the dimensions of the defect and the load-bearing mechanical stress differ substantially from those in a human joint. Thus, further research using larger animal models will better represent the true potential of these constructs.

Chen and co-workers [333] investigated exosome delivery using a 3D-printed, radially oriented gelatine methacrylate (GelMA)/dECM scaffold produced by DLP (Figure 13c) as a targeting strategy for the mitochondrial disfunction commonly associated with the pathophysiology of OC lesions. When implanted subcutaneously, the composite scaffold gave sustained exosome release over seven days and demonstrated high immunocompatibility and biodegradability. In a rabbit full-thickness OC defect, exosome-loaded GelMA/dECM scaffolds contributed to accelerated bone and cartilage regeneration over six weeks compared to blank GelMA/dECM scaffolds, GelMA only scaffolds, or untreated defects. At 12 weeks, however, there was no significant difference between the groups treated with GelMA/dECM constructs with or without exosomes. Notwithstanding this, exosome treatment led to a decrease in lesion-induced mitochondrial damage, resulting in lower mitochondrial vacuolation and reduced levels of malondialdehyde, an oxidative stress marker, in the joint synovial fluid [333]. It should be noted that exosome bioactivity after 3D printing was not assessed in comparison with freshly isolated exosomes; hence, it is not possible to know whether the 3D printing process had any detrimental effects on exosome biological action.

Therefore, despite the already established potential of standalone biomaterials, the regenerative power of a tissue-engineered scaffold can be greatly improved by the incorporation of cell-guiding biomolecular cues. Furthermore, it became evident that several aspects of the pathophysiology of an OC condition can be targeted when designing a therapeutic solution: the reduced stem cell potential within a lesion can be tackled by the delivery of chemotactic factors; sustained growth and differentiation factor delivery may circumvent the usual formation of fibrous tissue and promote the continuous deposition of hyaline cartilage instead; the pro-inflammatory milieu within a diseased joint can be shifted to an anti-inflammatory environment with the use of immunomodulators. Naturally, these cause-effect relationships are not as straightforward in practice as in theory. It is necessary to adjust the administration doses and tightly control the release profiles of the active ingredients, so as to maintain appropriate drug or protein levels at the defect site.

Accordingly, the release of bioactive elements from tissue-engineered constructs may be finely tuned by the use of multiple encapsulation strategies. For instance, using SLA, the release of TGF-β1 from 3D-printed scaffolds was further controlled by prior encapsulation of the growth factor within PLGA nanoparticles [334]. Likewise, Wen and colleagues incorporated free SDF-1α and microspheres loaded with the small molecule Y27632 into PU 3D-printed scaffolds, so as to achieve a faster release of the former and a controlled, slower release of the latter [151]. The early chemotactic action of SDF-1α would recruit progenitor cells to the defect site, which would then be differentiated to a chondrogenic phenotype by Y27632, a small molecule inhibitor of the ROCK signalling pathway previously shown to enhance chondrogenesis [335,336]. In fact, MSC migration and chondrogenic differentiation were promoted by SDF-1α and Y27632 in vitro, respectively. In vivo, even though dual-loaded scaffolds resulted in noticeable neotissue formation and ECM production compared to pristine PU scaffolds over 6 weeks, the newly formed cartilage lacked the structure and stratified organisation of native hyaline cartilage, which could perhaps be ameliorated at later time points after implantation [151].

Recent technological advancements have pushed AM even further in the direction of precision medicine in bone and cartilage therapy, with the establishment of in situ 3D printing techniques for OC defect repair [337,338]. In situ 3D printing in a clinical setting would involve, firstly, a high-resolution scan of the OC defect, which would then be converted into a CAD model of the scaffold to be fabricated. Finally, a portable 3D printing equipment would be used to print the digitally projected construct directly into the defect site. This methodology could shorten the time window that usually exists between scaffold manufacturing and implantation; moreover, the risk of contamination during scaffold preparation would be decreased [339]. The duration of the procedure can also be exceptionally short, taking up to one or two minutes for the whole 3D printing process [337,338]. In addition, in situ 3D bioprinting—that is, 3D printing of cell-laden bioinks—can also be carried, with excellent preservation of cell viability, secretion of ECM components, and neocartilage formation [340].

**Figure 14:**
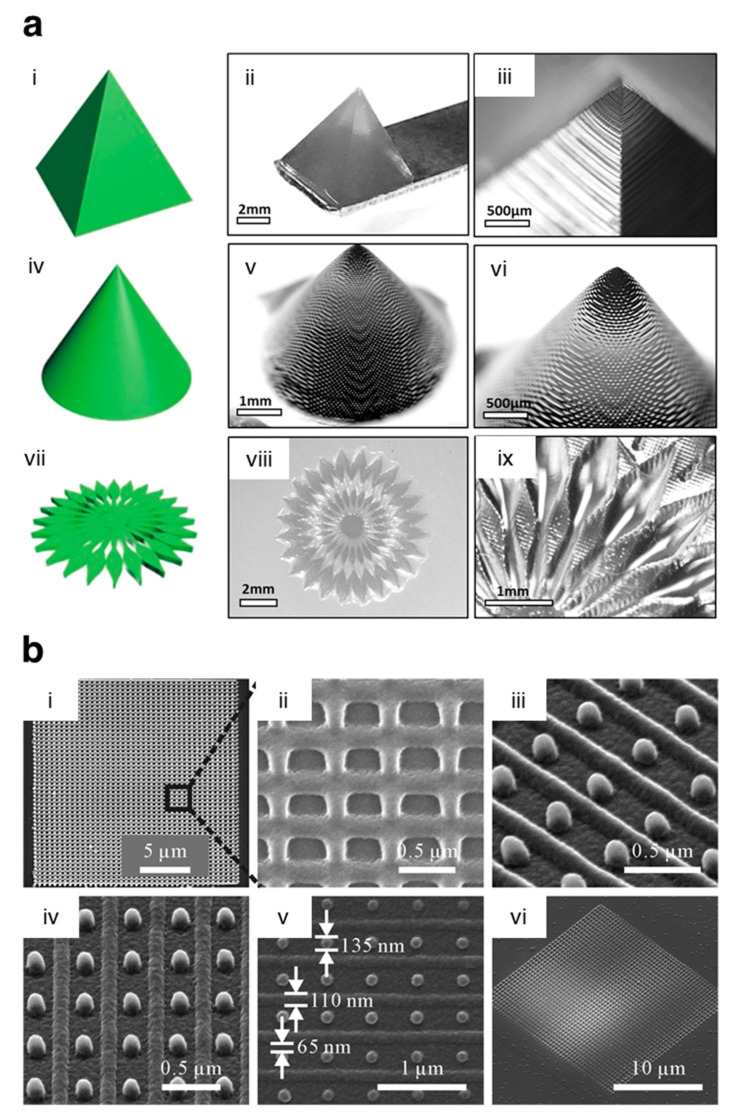
High-resolution 3D printing. (**a**) Solid hydrogel constructs generated by lithography-based biofabrication by DLP: (**i**–**iii**) pyramid, (**iv**–**vi**) cone, (**vii**–**ix**) flower with channels ranging from 50 μm to 500 μm. Adapted with permission from [342]. Copyright © 2018, IOP Publishing Ltd. (**b**) SEM images of high-resolution periodic structures fabricated via two-photon polymerisation, consisting of parallel lines and vertical pillars. By adjusting the laser focus during the printing process, it was possible to transition between undefined structures, in which the lines and the pillars were not distinguishable (**i**,**ii**), to well-defined periodic arrays, where the pillar and line organisation was well discernible (**iii**–**v**). (**vi**) displays the entire 3D-printed array. Adapted from [347] under the terms of the Creative Commons Attribution License (http://creativecommons.org/licenses/by/4.0/). Copyright © 2019, Zheng, et al.

However, AM is associated with a number of limitations that have been hindering its clinical applications. One of the major issues associated with traditional 3D printing techniques is very low resolution (>100 μm) which does not enable the consistent and reproducible manufacturing of micro- and nanoscaled devices [341]. High-resolution 3D printing methods, such as photopolymerisation-based 3D printing [342,343,344], have thus been developed as an answer to this problem, allowing the creation of 3D structures with increasingly complex architectures and exquisite micro- and nanofeatures (Figure 14). Particularly promising results have been achieved with laser two-photon polymerisation 3D printing, where submicron structures can be generated with a resolution under 100 nm (Figure 14b) [345,346,347]. Another disadvantage concerns the narrow range of materials that can be effectively printed: in extrusion-based methods, for instance, very specific rheological and mechanical properties need to be attained to allow for an adequate flow under shear stress and a stable layer-by-layer deposition [340]. Generally speaking, the list of biocompatible, cell-instructive materials that can be used in AM methodologies is limited [325]. It should also be noted that the typically high processing temperatures or laser-assisted polymerisation associated with several AM methodologies impair the encapsulation of thermolabile and laser-sensitive elements, such as proteins and cells, during the scaffold manufacturing process [348,349]. This is a very significant shortcoming of 3D printing techniques since it hinders the direct use of protein- or cell-loaded bioinks and forces the addition of such bioactive factors after each layer or whole scaffold fabrication. Lastly, the typically time-consuming layer-by-layer processing and the high costs associated with many AM techniques, especially those developed for high-resolution 3D printing, are currently prohibitive to their industrial implementation and mass production [341].

## 4. From Practice Back to Theory: What Separates the Promise of Tissue-Engineered Strategies from Clinical Success?

The extensive list of studies herein presented is proof that the treatment of OC pathological conditions remains challenging, relying still too greatly on palliative treatments rather than disease-modifying drugs and restorative therapies [186], and typically culminating in surgical joint replacement. The impact of OC diseases and lesions on an individual’s quality of life, as well as the economic burden associated with these conditions, emphasises the urgent need for the discovery and development of suitable alternatives. Taking the recent advancements in TE and cell-based therapy into account, why are the current OC therapies not satisfactory? What are the reasons behind the failure of initially promising treatment strategies? A major factor hindering the development of effective OC therapies is the complexity and inherent anisotropy of the OC unit, which makes it hard to generate biomimicking constructs and, therefore, efficiently emulate the natural architecture and ECM organisation of the joint. Although TE moved closer to offering a solution to this problem, the structural complexity and overall cohesion and mechanical stability that can be achieved in biofabricated scaffolds remain limited. In spite of the wide range of biomaterials that can be used in these constructs, it is still necessary to find a compromise between the cell-guiding behaviour typical of natural materials with the reproducibility and improved mechanical properties of synthetic materials. Biomedical manufacturing techniques such as electrospinning and 3D printing have allowed for undeniable progress in the field of OCTE, with the generation of numerous bioactive scaffolds capable of triggering regenerative responses and assisting tissue reorganisation and repair. Both electrohydrodynamic and AM techniques are associated with several drawbacks, but, from the studies presented in this work, it perhaps becomes clear that these limitations are somewhat complementary. Therefore, efforts have been made to combine both strategies and produce nanostructured 3D scaffolds. This can be done, for example, by introducing electrospun fibres into the inks used in conventional 3D printing methods, such as extrusion-based printing [350,351]. Alternatively, instead of performing electrospinning and 3D printing separately and sequentially, single-step electrohydrodynamic direct writing techniques have been developed. These encompass, for instance, NFES and MEW.

In a typical solution electrospinning process (Figure 7), charge accumulation at the tip of the spinneret leads to Taylor cone formation and ejection of the polymer solution. Initially, the jet follows a linear trajectory towards the grounded collector, but eventually, the effect of several electric and aerodynamic forces disrupts this linear course and causes the so-called bending or whipping instability [271]. This chaotic jet movement results in random fibre deposition, which hinders the construction of scaffolds with complex and customisable architectures. The advantage of NFES and MEW is based on the elimination of this whipping instability of the jet during electrospinning, enabling precise fibre deposition and fine control over the geometry of the construct. In the case of MEW, it is possible to surpass whipping instability due to the absence of solvents and the typically low conductivity and high melt viscosity of the polymers used [322]. In turn, NFES is performed at very short tip-to-collector distances (<5 cm) and low voltages, so that fibre deposition occurs within the linear trajectory of the electrified polymer jet [352]. Of note, whipping instability during electrospinning is critical for further fibre elongation and solvent evaporation, meaning that its suppression often results in fibres with larger diameters [352]. Nevertheless, recent studies have proven the potential of electrohydrodynamic direct writing techniques in the field of cartilage/osteochondralTE [353,354].

Recently, AM methodologies also developed to enable scaffold manufacturing in four dimensions, where the fourth dimension is that of time, giving rise to 4D printing. 4D printing makes use of common 3D printing techniques to generate shape-shifting materials, where the original geometry can dynamically change upon application of an external stimulus, such as temperature [355], osmotic pressure [356], or a magnetic field [357]. The possibility of generating materials capable of accompanying the natural dynamic behaviour of biological tissues holds great promise in the field of regenerative medicine. Even though to the best of the authors’ knowledge, no studies have yet been published applying 4D printing to OCTE, both tracheal cartilage [356] and cancellous bone [358] scaffolds have recently been created with this technology, demonstrating exceptional mechanical properties, no toxicity, and promising in vivo performance.

Because in vivo testing is mandatory in the development of medical products, a suitable choice of animal models is imperative to guarantee some level of parallelism to human conditions. Several criteria must be considered when making this decision, including the type and location of the OC defect, the dimensions of the joint and cartilage thickness, and the stage of skeletal development of the animals [359]. The first in vivo experiments are always performed in smaller animals, usually rodents (mice, rats, guinea pigs) and rabbits, due to lower costs and simpler handling and maintenance conditions [360]. However, these animals are not perfect models to study human OC repair and regeneration, owing to the small size of their articulations and differences in terms of regenerative potential and joint load distribution [361]. As such, in pre-clinical trials, it is necessary to test the therapeutic products in larger animal models, including sheep, goats, pigs, and horses, which can better resemble the anatomy and physiology of human joints. The use of larger animals in scientific experiments is, however, associated with extensive economic, logistic, and ethical hurdles, and these considerations are an important factor when designing in vivo experiments [361]. Of note, the animal sample size in pre-clinical trials can be greatly reduced with the development of suitable in vitro and ex vivo models with human cells and tissues that can effectively recapitulate the pathophysiological environment of OC disease [359].

Finally, the clinical trials and regulatory obstacles that need to be overcome before a new product is applied clinically and commercialised, along with the inevitable high costs associated, are another critical aspect that contributes to an extended time-to-market [362]. Although there are some promising examples of tissue-engineered OC products available on the market (as thoroughly reviewed by [42,363]), their use is still not consensual and widespread among the orthopaedic medical community. Their design is often based on bi/triphasic cell-free scaffold strategies that cannot fully replicate the native complexity of the OC unit and thereby struggle to achieve functional repair and regeneration of damaged OC tissue. Furthermore, it should be taken into consideration that the extension, shape, and depth of OC lesions is highly variable between different individuals, implying that “one size fits all” strategies are often not ideal. This is where personalised medicine represents a valuable tool to develop patient-specific solutions.

Further technological advancements not only in the field of biofabrication but also in medical imaging and 3D modelling, will allow for the development of therapeutic scaffolds with structural and chemical characteristics closer to those observed within an OC unit. Of note, apart from achieving clinical safety and efficacy, it is also pivotal to ensure that the production of such tissue-engineered scaffolds is scalable for industrial manufacturing under cGMP and economically viable, which is still a major challenge when using cell-based therapy or constructs with intricate architecture and chemical composition. Unfortunately, it is still difficult, even using current laboratory scale techniques, to achieve artificial scaffolds with physiologically relevant sizes and appropriate micro-/nanostructures, not only to mimic the natural architecture of the ECM but also other elements such as the blood vessels present in the joint. This is especially relevant in OCTE constructs, given the lack of vascularisation of AC and its dependence on surrounding tissues for nutritional support.

Thus, it is possible to conclude that the development of OCTE products is inevitably subjected to close scrutiny to ensure safety and efficacy as well as logistic and economic viability. The ideal OCTE scaffold will, hence, sustain long-term OC regeneration, promoting progenitor cell homing, differentiation, and replacement of the scaffold matrix with spatially organised bone and cartilage neotissue, while exerting no toxic effects. Moreover, it is important to ensure that the production costs will not be prohibitive for universal access to the therapy, and that large-scale manufacturing and supply will be able to meet the overall demand for OC treatment solutions.

## 5. Conclusions

OC diseases and traumatic lesions affect a progressively greater portion of the world’s population, with considerable impacts on quality of life and significant economic burdens. Current treatment strategies are mainly palliative and have short-term efficacy, highlighting the need for therapeutic options capable of halting disease progression, ameliorating joint pain and mobility, and stimulating tissue regenerative responses. TE offers a means of generating biomimicking platforms able to reproduce the natural geometry and microarchitecture of the OC unit, while delivering progenitor cells and/or cell-guiding cues (e.g., growth factors, small molecule drugs) to the lesion site. Electrospinning and AM are two types of manufacturing technology widely used for biofabrication of scaffolds with varying composition and structure, whose potential for OCTE has been demonstrated in several in vitro and in vivo studies. However, to date, no therapeutic strategy has been developed that can sustain long-term tissue regeneration and satisfactory functional recovery. On one hand, this is due to the structural, chemical, and biological complexity of the OC unit, which cannot be easily reproduced artificially; on the other hand, the absence of standardised protocols for scaffold production, biomolecule administration, and therapeutic outcome evaluation obstructs reproducibility and comparison among different products. The development of an OCTE solution capable of responding to these issues is therefore dependent not only on technological progress, but also on strengthened communication between researchers and protocol uniformisation to achieve robust, reproducible, and scalable tissue-engineered solutions.

## Figures and Tables

**Table 2:** Summary of the main cell types used for OCTE, with their advantages and disadvantages.

Cell Type			Advantages	Disadvantages
**Pluripotent**	Embryonic Stem Cells (ESCs)		High differentiation and self-renewal capacity; Off-the-shelf source	Ethical concerns; Tumorigenic potential and genomic instability; Heterogeneous differentiation
	Induced Pluripotent Stem Cells (iPSCs)		High differentiation and self-renewal capacity; Patient-specific therapy; Minimally invasive harvest technique for autologous iPSCs; Off-the-shelf source	Tumorigenic potential and genomic instability; Difficulty in achieving uniform differentiation; High cost
**Multipotent**	Mesenchymal Stem Cells (MSCs)	Bone Marrow-Derived Stem Cells (BMSCs)	High chondrogenic and osteogenic potential	Invasive harvest technique; Low collection yields force them to be heavily expanded before sufficient numbers are attained (longer waiting times and higher risk of de-differentiation); Differentiation potential declines with increasing age Possibility of forming heterogeneous cell populations
		Adipose-Derived Stem Cells (ASCs)	Minimally invasive isolation procedure with high yields	Lower chondrogenic and osteogenic potential than BMSCs
		Emerging MSC types: synovial tissue MSCs (SMSCs), periosteum-derived MSCs (PMSCs), umbilical cord MSCs (UCMSCs), amniotic membrane and fluid MSCs (AFSCs)		
**Unipotent**	Primary cells (chondrocytes and osteoblasts)		Native phenotype; No need for osteogenic/chondrogenic differentiation protocols; Easy accessibilityImmunocompatibility (autologous sources)	Limited lifespan; Low proliferation potential; Risk of de-differentiation or loss of function during expansion; Limited cell numbers obtained during isolation; Risk of donor-site morbidity and infection upon autologous cell isolation
